# Association between metabolic syndrome and risk of incident dementia in UK Biobank

**DOI:** 10.1002/alz.13439

**Published:** 2023-09-07

**Authors:** Danial Qureshi, Jennifer Collister, Naomi E. Allen, Elżbieta Kuźma, Thomas Littlejohns

**Affiliations:** ^1^ Nuffield Department of Population Health University of Oxford Oxford UK; ^2^ UK Biobank Ltd Stockport UK; ^3^ Albertinen Haus Centre for Geriatrics and Gerontology University of Hamburg Hamburg Germany

**Keywords:** cohort studies, dementia, follow‐up studies, incidence, longitudinal, metabolic syndrome, risk factors, UK biobank

## Abstract

**INTRODUCTION:**

The association between metabolic syndrome (MetS) and incident dementia remains inconclusive.

**METHODS:**

In 176,249 dementia‐free UK Biobank participants aged ≥60 years at baseline, Cox proportional‐hazards models were used to investigate the association between MetS and incident dementia. MetS was defined as the presence of ≥3 of the following: elevated waist circumference, triglycerides, blood pressure, blood glucose, and reduced high‐density lipoprotein cholesterol.

**RESULTS:**

Over 15 years of follow‐up (median = 12.3), 5255 participants developed dementia. MetS was associated with an increased risk of incident dementia (hazard ratio [HR]: 1.12, 95% confidence interval [CI]: 1.06, 1.18). The association remained consistent when restricting to longer follow‐up intervals: >5 to 10 years (HR: 1.17, 95% CI: 1.07, 1.27) and >10 years (HR: 1.22, 95% CI: 1.12, 1.32). Stronger associations were observed in those with ≥4 MetS components and in apolipoprotein‐E (*APOE*)‐ε4 non‐carriers.

**DISCUSSION:**

In this large population‐based prospective cohort, MetS was associated with an increased risk of dementia.

**Highlights:**

- MetS was associated with a 12% increased risk of incident all‐cause dementia.
- Associations remained similar after restricting the analysis to those with longer follow‐up.
- The presence of four or five MetS components was significantly associated with dementia.
- Stronger associations were observed in those with a low genetic risk for dementia.

## 1 BACKGROUND

Approximately 20% to 25% of adults globally live with metabolic syndrome (MetS), a condition characterized by the clustering of several cardiometabolic abnormalities.^1^ MetS is diagnosed based on the presence of at least three of the following: elevated (1) waist circumference, (2) triglycerides, (3) blood pressure, (4) and blood glucose and (5) reduced high‐density lipoprotein (HDL) cholesterol.^1^ The prevalence of MetS increases with age; in the US, an estimated 40% of individuals aged ≥60 years meet the diagnostic criteria.^2^, ^3^ MetS is strongly associated with an increased risk of developing cardiovascular disease, cerebrovascular disease, and Type 2 diabetes.^1^

Recent research has indicated that MetS could represent a novel risk factor for dementia; however, the relationship remains unclear. A 2019 meta‐analysis of six longitudinal studies found no statistically significant pooled association between MetS and risk of incident dementia (hazard ratio [HR]: 1.12, 95% confidence interval [CI]: 0.94, 1.33).^4^ Notably, two additional longitudinal studies have since been published, providing further insight regarding the relationship between MetS and dementia risk, both of which reported an increased risk of dementia of 10% to 15% for those with MetS, although only one study reached statistical significance.^5^, ^6^ As such, the relationship between MetS and dementia remains inconclusive, with the majority of studies consisting of small sample sizes (i.e., <8000 participants^5^, ^7^, ^8^, ^9^, ^10^, ^11^, ^12^, ^13^, ^14^) or short follow‐up periods (ie, <5 years^6^, ^8^, ^10^, ^11^, ^12^, ^13^, ^14^). The latter is important to address because dementia has a long prodromal phase prior to a clinical diagnosis, and reverse causation is a particular limitation of studies with short follow‐up. Furthermore, previous studies demonstrated that associations between individual MetS components (eg, elevated blood glucose, blood pressure or waist circumference) and cognitive dysfunction and/or dementia may differ by genetic risk for dementia.^15^, ^16^, ^17^, ^18^, ^19^ However, whether genetic predisposition to dementia modifies any observed associations between MetS and dementia risk has, to our knowledge, not been previously investigated. Moreover, previous evidence indicates that, individually, each component of MetS is consistently associated with an increased risk of developing dementia.^20^ However, the combined contribution of these components in this relationship is not fully understood.

To address these limitations, we investigated the association between MetS and risk of incident dementia in a population‐based cohort of more than 175,000 participants over 15 years of follow‐up. We also examined whether Apolipoprotein‐E (*APOE*)‐ε4 carrier status (either *APOE* ε3/ε4 or *APOE* ε4/ε4), as well as a non‐*APOE* polygenic risk score for dementia, interacts with MetS to modify the risk of dementia. Additionally, we further explored the role of MetS components on dementia risk.

**RESEARCH IN CONTEXT**

1. **Systematic review**: A PubMed search identified studies investigating the relationship between metabolic syndrome (MetS) and dementia. Findings remain inconclusive, with most studies conducted in small populations with short follow‐up duration.
2. **Interpretation**: In a large population‐based study of more than 175,000 participants aged ≥60 at study baseline, we found that MetS was associated with a 12% increased risk of incident all‐cause dementia. The associations remained similar when restricting the analysis to those with longer follow‐up. Stronger associations were observed in those with at least four MetS components and in Apolipoprotein‐E (*APOE*)‐ε4 non‐carriers. The relative risk for dementia was greater among *APOE*‐ε4 non‐carriers with MetS. However, the absolute risk difference between those with and without MetS was larger among ε4 carriers.
3. **Future directions**: These findings should be replicated in other large and diverse cohorts with long follow‐up duration. Differential associations between MetS and dementia subtypes, such as vascular dementia and Alzheimer's disease, should also be further investigated.

## 2 METHODS

### 2.1 Study population

The UK Biobank is a large population‐based prospective cohort study of more than half a million participants aged 40 to 69 years recruited between 2006 and 2010 in the UK.^21^ Participants attended baseline assessment centers across England, Scotland, and Wales, where they provided electronically signed consent. At baseline, participants provided information on sociodemographic, lifestyle, environmental, and health‐related factors collected through a touch‐screen questionnaire and a nurse‐led verbal interview, underwent various physical examinations, and provided biological samples. Medication use was also ascertained during a nurse‐led verbal interview. UK Biobank received ethical approval from the National Health Service (NHS) North West Centre for Research Ethics Committee (Ref: 11/NW/0382).

In the current study, the sample was restricted to participants aged ≥60 years at baseline to ensure the study population consisted of individuals who were at risk of developing late‐onset dementia. We also excluded participants with prevalent self‐reported or diagnosed dementia. Further details regarding cohort creation are provided in Supplemental File 1.

### 2.2 Metabolic syndrome

MetS was defined using the Harmonized Criteria proposed by the International Diabetes Federation (IDF) and the American Heart Association/National Heart, Lung, and Blood Institute (AHA/NHLBI) in 2009.^1^ The presence of at least three of the following five components constituted a MetS diagnosis: (1) abdominal obesity (elevated waist circumference: ≥102 cm in males and ≥88 cm in females); (2) elevated triglycerides (≥150 mg/dL or 1.7 mmol/L); (3) elevated blood pressure (≥130 mmHg systolic blood pressure and/or ≥85 mmHg diastolic blood pressure) or antihypertensive medication use; (4) elevated fasting blood glucose (≥100 mg/dL or ≥5.6 mmol/L) or drug treatment for elevated blood glucose; and (5) reduced HDL‐cholesterol (<40 mg/dL or 1.0 mmol/L in males; <50 mg/dL or 1.3 mmol/L in females) or lipid‐modifying medications. Data on circulating glucose levels were obtained predominantly from non‐fasting blood samples, which are more likely to be affected by recent food intake (compared to fasting samples), which can lead to high variability in glucose measurements. Therefore, we used glycated hemoglobin (HbA1c) as a proxy measure of glucose, based on the recommendations of the American Diabetes Association, with a cut point of HbA1c ≥5.7% (39 mmol/mol) to represent hyperglycemia.^22^ Additionally, lipid‐modifying medications are known to have multiple effects on the lipid profile (including HDL cholesterol and triglycerides).^23^ Thus, to prevent double counting, we assigned these medications to the reduced HDL‐cholesterol group in our analysis. Medication usage was captured using Anatomical Therapeutic Chemical (ATC) codes, informed by a thorough literature review of previous studies defining MetS using ATC codes,^24^, ^25^, ^26^, ^27^, ^28^, ^29^, ^30^, ^31^, ^32^, ^33^, ^34^ and with expert clinical input.

Participants were categorized into two groups: (1) no MetS (reference group) and (2) MetS. Complete information regarding the variables and medication codes used to capture and define MetS components are available in Supplemental Files 2 and 3.

### 2.3 Dementia

Dementia cases were identified using hospital inpatient admissions and death registry records. Inpatient admissions records were available from the Hospital Episode Statistics for England, the Scottish Morbidity Record for Scotland, and the Patient Episode Database for Wales. Death registry records were available from the NHS England for England and Wales, and the Information and Statistics Division for Scotland. Primary and secondary hospital diagnoses and causes of death were recorded using the International Classification of Diseases (ICD‐10) coding system. The ICD codes used to ascertain dementia were selected and validated by the UK Biobank outcome adjudication group (Supplemental File 4).^35^

### 2.4 Covariates and effect modifiers

Covariates included sociodemographic, lifestyle, and genetic factors previously associated with dementia or MetS and are considered here to be potential confounders in determining the relationship between the exposure and outcome.^20^, ^36^ Age (in years) at baseline was calculated based on date of birth and the date of attending an assessment center. The Townsend deprivation index score was used as a proxy for material socioeconomic deprivation and was assigned to each study participant using their residential postal code at baseline, and was categorized in fifths (1 to 5: least deprived to most deprived).^37^ Sex (“male,” “female”) was captured using NHS records and/or self‐reported data from the touch‐screen questionnaire. Ethnicity (“white,” “non‐white”), education level (“primary,” “secondary,” “post‐secondary non‐tertiary,” “tertiary”), household income in GBP (“less than 18,000,” “18,000 to 30,999,” “31,000 to 51,999,” “52,000 to 100,000,” “greater than 100,000”), smoking status (“never,” “previous,” “current”), and alcohol intake (“never,” “former drinker,” “special occasions only,” “1 to 3 times per month,” “1 or 2 times per week,” “3 or 4 times per week,” “daily or almost daily,” “prefer not to answer”) were captured from the touch‐screen questionnaire. Physical activity level (“low” – metabolic equivalent [MET] minutes ≤1200, “high” – MET > 1200) was derived from the touch‐screen questionnaire items, which were adapted to the validated short International Physical Activity Questionnaire^38^; time spent conducting vigorous, moderate, and walking activity was weighted by the amount of energy expended, which allowed us to obtain total MET minutes per week.

*APOE*‐ε4 carrier status (*“*ε4 non‐carrier,” *“*ε4 carrier”) was derived using the rs429358 and rs7412 single‐nucleotide polymorphisms (SNP), which were directly genotyped on the UK Biobank arrays.^39^ Non‐*APOE* dementia polygenic risk score (PRS) was used in secondary analyses to assess interactions. This measure was based on a 39 SNP score (available on the Polygenic Score Catalog online as PGS001775) and was derived based on previously published methods^40^ that used a range of publicly available genome‐wide association study summary statistics for late‐onset Alzheimer's disease.^41^, ^42^, ^43^ Weights for the score were computed in the International Genomics of Alzheimer's Project.^42^, ^43^ PLINK 2^44^ with a hard call threshold of 0.1 was used to make sure that none of the SNPs were in linkage disequilibrium with the *APOE* SNPs (*R^2^* < 0.3). One SNP had minor allele frequency <0.005 and was therefore excluded. The final score was made up of 38 SNPs, where all SNPs had imputation information >0.9 and no SNPs were ambiguous. The dementia PRS was split into quintiles and further categorized into “low” (quintile 1), “intermediate” (quintiles 2 to 4), and “high” (quintile 5) groups, with a higher PRS indicating a greater dementia risk.

### 2.5 Statistical analysis

Descriptive statistics were used to compare baseline characteristics between participants with and without MetS; mean and standard deviation was calculated for normally distributed variables, and median and interquartile range for skewed variables. Multivariable Cox proportional‐hazards models using follow‐up time as the underlying time scale were used to estimate the association between MetS and incident dementia. Follow‐up time (in years) was calculated from the baseline assessment date until the date of first incident dementia diagnosis, date of death, date of loss to follow‐up, or end of follow‐up, whichever occurred first. End of complete follow‐up was based on the availability of electronic health record data in the UK Biobank, which was censored on September 30, 2021 for England; July 31, 2021 for Scotland; and February 28, 2018 for Wales. The proportional‐hazards assumption was visually examined using scaled Schoenfeld residuals, with no variables violating the assumption. Only participants with complete data on all five individual MetS components were included in the main analysis (see Supplemental File 1 for cohort flow diagram). The main analysis was adjusted for age, sex, ethnicity, education, Townsend deprivation index score, household income, smoking status, alcohol intake, physical activity level, and *APOE*‐ε4 carrier status.

Participants with any missing covariate data or who responded with “prefer not to answer/do not know” were assigned as a separate category for each categorical variable.

To investigate the potential for reverse causation, the analysis was stratified by follow‐up time: (1) ≤5 years, (2) >5 to 10 years, and (3) ≥10 years.

To better understand the influence of each confounder on the relationship between MetS and risk of incident dementia, we investigated the effect of individual and sequential adjustment of covariates. In individual adjustment, we first adjusted for age and sex and then incorporated each covariate into the model separately. In sequential adjustment, we again adjusted for age and sex and then gradually added all covariates into the model in a stepwise manner.

The interaction between MetS and genetic predisposition to dementia was investigated by entering MetS × *APOE*‐ε4 carrier status (“not a carrier,” “ε4 carrier”) MetS × non‐*APOE* dementia PRS (“low,” “intermediate,” “high”) interaction terms separately into the main model. Effect estimates within each strata of genetic risk were obtained for MetS versus no MetS.

To investigate possible joint effects by MetS and genetic predisposition to dementia, a four‐level categorical variable was derived containing each combination of MetS and *APOE*‐ε4 carrier status; a six‐level categorical variable was also derived containing each combination of MetS and non‐*APOE* dementia PRS categories. The main analysis was repeated with these variables entered into separate models. This approach provides a comprehensive understanding of how these individual genetic factors may synergistically influence dementia risk by MetS status.

The interaction between MetS and sex (“female,” “male”) was also examined due to prior evidence of effect modification.^45^, ^46^ Furthermore, we conducted stratified analyses to investigate effect modification by age group.

We also examined the association between individual MetS components and dementia risk, as well as the association between the number of MetS components (defined as a categorical variable, on a scale from 0 to 5) and dementia risk. Additionally, we investigated the relationship between all 16 possible combinations of MetS components and incident dementia.

In our study, we assigned lipid‐modifying medications to the reduced HDL cholesterol group. To explore whether this decision impacted our findings, we further examined the effect of assigning these medications to the elevated triglycerides group instead of reduced‐HDL cholesterol.

We also performed sensitivity analyses to examine the robustness of the findings, including repeating the main analysis with (1) the use of age as the underlying time scale; (2) exclusion of participants with shorter follow‐up time to avoid potential differential bias; (3) accounting for death as a competing risk; (4) additional adjustment for cardiovascular disease (“yes,” “no”) – defined as a diagnosis of stroke, coronary heart disease, or heart failure captured from the nurse‐led verbal interview (which may be a confounder or on the causal pathway)^1^; (5) using the NCEP‐ATP III definition for MetS,^47^ which is a commonly used alternative definition for the condition; and (6) using multiple imputation to investigate the potential impact of missing data (detailed description provided in Supplemental File 5);

All *p* values were two sided, with statistical significance set at *p* < .05. All analyses were performed using RStudio version 4.2.2.

## 3 RESULTS

Among 502,414 participants recruited into UK Biobank, we excluded 284,951 participants aged <60 years, 166 with prevalent dementia, and 41,048 with missing data on ≥1 MetS components. Baseline characteristics of participants with missing data on MetS components were highly similar to those with complete data (Supplemental File 6). The final analytical sample consisted of 176,249 participants, of whom 41.7% had prevalent MetS. Participants’ characteristics according to incident dementia status at follow‐up are presented in Supplementary File 7.

The baseline characteristics of the study participants are provided in Table 1. Compared to participants without MetS, those with MetS were more likely to be older, male, of non‐white ethnicity, have lower educational qualifications, reside in more socioeconomically deprived areas, have lower household income levels, be current/previous smokers, be less physically active, and be *APOE*‐ε4 carriers. Among those with MetS, 52.8% had three, 33.2% had four, and 14.0% had five MetS components; the most prevalent component being elevated blood pressure (96.2%), followed by elevated triglycerides (73.5%), reduced HDL cholesterol (71.9%), elevated waist circumference (69.8%), and elevated HbA1c (49.8%). Over 2,088,296 person‐years of follow‐up (median [interquartile range]: 12.3 [11.5 to 13.1] years), 5255 cases of incident all‐cause dementia were identified.

**TABLE 1:** Baseline characteristics by metabolic syndrome (MetS) status.

Characteristic		No MetS(*N* = 102739)	MetS (*N* = 73510)	Overall (*N* = 176249)
Follow‐up length	Mean (SD)	12.0 (2.0)	11.7 (2.4)	11.8 (2.2)
Age (years)	Mean (SD)	64.0 (2.8)	64.4 (2.9)	64.1 (2.9)
Sex	Female	56,323 (54.8%)	35,729 (48.6%)	92,052 (52.2%)
	Male	46,416 (45.2%)	37,781 (51.4%)	84,197 (47.8%)
Ethnicity	White	100,305 (97.6%)	70,369 (95.7%)	170,674 (96.8%)
	Non‐White	1987 (1.9%)	2777 (3.8%)	4764 (2.7%)
	Missing	447 (0.5%)	364 (0.5%)	811 (0.5%)
Education level	Primary	23,907 (23.3%)	23,318 (31.7%)	47,225 (26.8%)
	Secondary	48,010 (46.7%)	29,675 (40.4%)	77,685 (44.1%)
	Post‐secondary non‐tertiary	11,261 (11.0%)	7661 (10.4%)	18,922 (10.7%)
	Tertiary	18,329 (17.8%)	11,692 (15.9%)	30,021 (17.0%)
	Missing	1232 (1.2%)	1164 (1.6%)	2396 (1.4%)
Townsend deprivation index, quintiles	1 (least deprived)	22,162 (21.6%)	13,059 (17.8%)	35,221 (20.0%)
	2	21,438 (20.9%)	13,782 (18.7%)	35,220 (20.0%)
	3	20,989 (20.4%)	14,231 (19.4%)	35,220 (20.0%)
	4	20,182 (19.6%)	15,038 (20.5%)	35,220 (20.0%)
	5 (most deprived)	17,882 (17.4%)	17,338 (23.6%)	35,220 (20.0%)
	Missing	86 (0.1%)	62 (0.1%)	148 (0.1%)
Household income (in GBP)	Less than 18,000	25,097 (24.4%)	23,588 (32.1%)	48,685 (27.6%)
	18,000 to 30,999	27,809 (27.1%)	18,821 (25.6%)	46,630 (26.5%)
	31,000 to 51,999	18,982 (18.5%)	10,954 (14.9%)	29,936 (17.0%)
	52,000 to 100,000	9589 (9.3%)	4848 (6.6%)	14,437 (8.2%)
	Greater than 100,000	2351 (2.3%)	1058 (1.4%)	3409 (1.9%)
	Missing	18,911 (18.4%)	14,241 (19.4%)	33,152 (18.8%)
Smoking status	Never	54,640 (53.2%)	32,645 (44.4%)	87,285 (49.5%)
	Previous	40,038 (39.0%)	33,354 (45.4%)	73,392 (41.6%)
	Current	7563 (7.4%)	6945 (9.4%)	14,508 (8.2%)
	Missing	498 (0.5%)	566 (0.8%)	1064 (0.6%)
Alcohol intake	Never	4038 (3.9%)	4392 (6.0%)	8430 (4.8%)
	Former drinker	3176 (3.1%)	3466 (4.7%)	6642 (3.8%)
	Special occasions only	10,253 (10.0%)	11,030 (15.0%)	21,283 (12.1%)
	1‐3 times per month	9377 (9.1%)	8024 (10.9%)	17,401 (9.9%)
	1‐2 times per week	24,086 (23.4%)	17,992 (24.5%)	42,078 (23.9%)
	3‐4 times per week	24,691 (24.0%)	14,204 (19.3%)	38,895 (22.1%)
	Daily or almost daily	26,978 (26.3%)	14,227 (19.4%)	41,205 (23.4%)
	Prefer not to answer	77 (0.1%)	91 (0.1%)	168 (0.1%)
	Missing	63 (0.1%)	84 (0.1%)	147 (0.1%)
Physical activity level	Low (MET minutes ≤1200)	24,873 (24.2%)	22,992 (31.3%)	47,865 (27.2%)
	High (MET minutes >1200)	57,557 (56.0%)	33,502 (45.6%)	91,059 (51.7%)
	Missing	20,309 (19.8%)	17,016 (23.1%)	37,325 (21.2%)
*APOE*‐ε4 carrier status	Non‐carrier	73,825 (71.9%)	51,909 (70.6%)	125,734 (71.3%)
	Carrier	25,686 (25.0%)	19,219 (26.1%)	44,905 (25.5%)
	Missing	3228 (3.1%)	2382 (3.2%)	5610 (3.2%)
Elevated waist circumference	Present	14,731 (14.3%)	51,275 (69.8%)	66,006 (37.5%)
Elevated triglycerides	Present	23,094 (22.5%)	54,002 (73.5%)	77,096 (43.7%)
Elevated blood pressure^a^	Present	77,127 (75.1%)	70,692 (96.2%)	147,819 (83.9%)
Elevated HbA1c^a^	Present	7895 (7.7%)	36,636 (49.8%)	44,531 (25.3%)
Reduced HDL‐cholesterol^a^	Present	15,791 (15.4%)	52,888 (71.9%)	68,679 (39.0%)

*Note*: Percentages do not add up to 100 due to rounding.

Abbreviations: APOE, apolipoprotein; GBP, British pound sterling; HbA1c, *hemoglobin A1c*; HDL, high‐density lipoprotein; MET, metabolic equivalent of task; PRS, polygenic risk score; SD, standard deviation.

^a^ Includes medication use.

**FIGURE 1:**
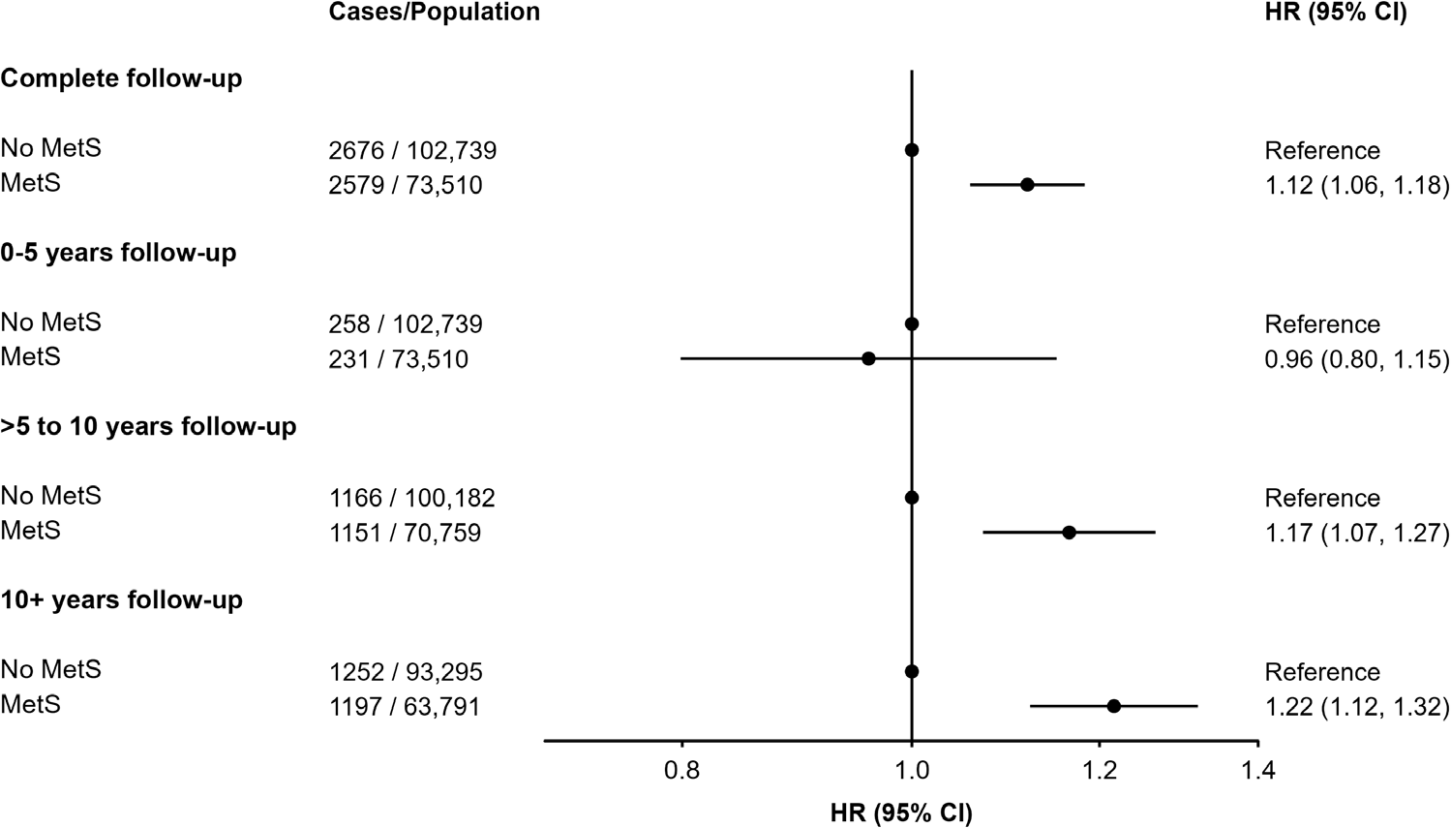
Cox proportional hazards models investigating the association between metabolic syndrome (MetS) and incident dementia by different follow‐up periods. HR = hazard ratio, CI = confidence interval, Ref. = reference group, IQR = interquartile range. Mean, median (IQR) follow‐up length in years: (1) 0–5 years: 4.9, 5.0 (5.0, 5.0); (2) >5 to 10 years: 9.8, 10.0 (10.0, 10.0); (3) 10+ years: 12.5, 12.5 (11.9, 13.2). Mean age of participants in years: (1) 0 to 5 years: 64.1; (2) >5 to 10 years: 64.1; (3) 10+ years: 64.0 years. Model adjusted for age, sex, ethnicity, Townsend deprivation index, education, household income, smoking status, alcohol intake, physical activity, and APOE‐ε4 carrier status.

Compared to participants with no MetS, those with MetS had an increased risk of incident all‐cause dementia (fully adjusted HR: 1.12, 95% CI: 1.06, 1.18, Figure 1 and Supplemental File 8). The association remained similar when restricting the analysis to >5 to 10 years (HR: 1.17, 95% CI: 1.07, 1.27) and >10 years of follow‐up (HR: 1.22, 95% CI: 1.12, 1.32), but was null for those with ≤5 years of follow‐up (HR: 0.96, 95% CI: 0.80, 1.15). In sensitivity analyses, the results remained similar after (1) using age as a time scale, (2) excluding participants who had a shorter follow‐up time (i.e., those from Wales), (3) accounting for death as a competing risk, (4) using the NCEP‐ATP III definition for MetS, and (5) performing multiple imputation for missing exposure and covariate data (see Supplemental File 9). While additional adjustment for cardiovascular disease did not change the direction of the association between MetS and dementia, the relationship was slightly attenuated (HR: 1.05, 95% CI: 1.01, 1.13).

There was no statistical evidence of an interaction between MetS and sex and risk of incident dementia (*p* value for interaction: .33; see Supplemental File 10). Age‐stratified analyses demonstrated that the strength of the association between MetS and dementia was slightly greater in those aged <65 years compared to older participants, but the direction was the same (Supplemental File 11).

There was statistical evidence of an interaction between MetS and *APOE*‐ε4 carrier status (*p* value for interaction: <.001) on the risk of incident dementia (Table 2). MetS was associated with dementia risk in *APOE*‐ε4 non‐carriers (HR: 1.26, 95%CI: 1.16, 1.37) but not in *APOE*‐ε4 carriers (HR: 1.02, 95%CI: 0.94, 1.10). However, despite higher relative risks in non‐carriers, the absolute incidence of dementia was higher in *APOE*‐ε4 carriers (Supplemental File 12). Specifically, among non‐carriers, the 12‐year cumulative dementia incidence was 2.3% (95% CI: 2.20, 2.40) for those with MetS and 1.4% (95% CI: 1.30, 1.50) for those without MetS. In comparison, among *APOE*‐ε4 carriers, the corresponding incidence was 6.5% (95% CI: 6.20, 6.90) for those with MetS and 5.1% (95% CI: 4.80, 5.40) for those without MetS. Therefore, the risk difference was higher for *APOE‐*ε4 carriers (1.4%) than non‐carriers (0.9%). No statistically significant interaction was found between MetS and non‐*APOE* dementia PRS (*p* value for interaction: .42) and risk of incident dementia (Table 2). Results from joint effects models are provided in Supplemental File 13.

**TABLE 2:** Cox proportional‐hazards models investigating the association between metabolic syndrome (MetS) and incident dementia by genetic predisposition.

Genetic factor	Cases/population	HR (95%CI)
**(A) APOE‐ε4 carrier status**		
Non‐carrier		
No MetS	1152/73,825	1 (Ref.)
MetS	1230/51,909	1.26 (1.16, 1.37)
Carrier		
No MetS	1424/25,686	1 (Ref.)
MetS	1261/19,219	1.02 (0.94, 1.10)
**(B) Non‐APOE dementia PRS**		
Low		
No MetS	297/16,759	1 (Ref.)
MetS	337/12,255	1.19 (1.02, 1.40)
Intermediate		
No MetS	1272/50,852	1 (Ref.)
MetS	1225/36,188	1.12 (1.04, 1.22)
High		
No MetS	594/17,159	1 (Ref.)
MetS	557/11,854	1.13 (1.01, 1.28)

Abbreviations: APOE, apolipoprotein E; CI, confidence interval; HR, hazard ratio; MetS, metabolic syndrome; PRS, polygenic risk score; Ref., reference group.

(A) Model adjusted for age, sex, ethnicity, Townsend deprivation index, education, household income, smoking status, alcohol intake, and physical activity. Excluded: 5,610 with missing information on APOE‐ε4 carrier status. *P* value for overall interaction between MetS and APOE‐ε4: < 0.001.

(B) Model adjusted for age, sex, ethnicity, Townsend deprivation index, education, household income, smoking status, alcohol intake, physical activity, and APOE‐ε4 carrier status. Excluded: 31,182 with missing information on non‐APOE dementia PRS. *P* value for overall interaction between MetS and non‐APOE dementia PRS: 0.42.

**FIGURE 2 alz13439-fig-0002:**
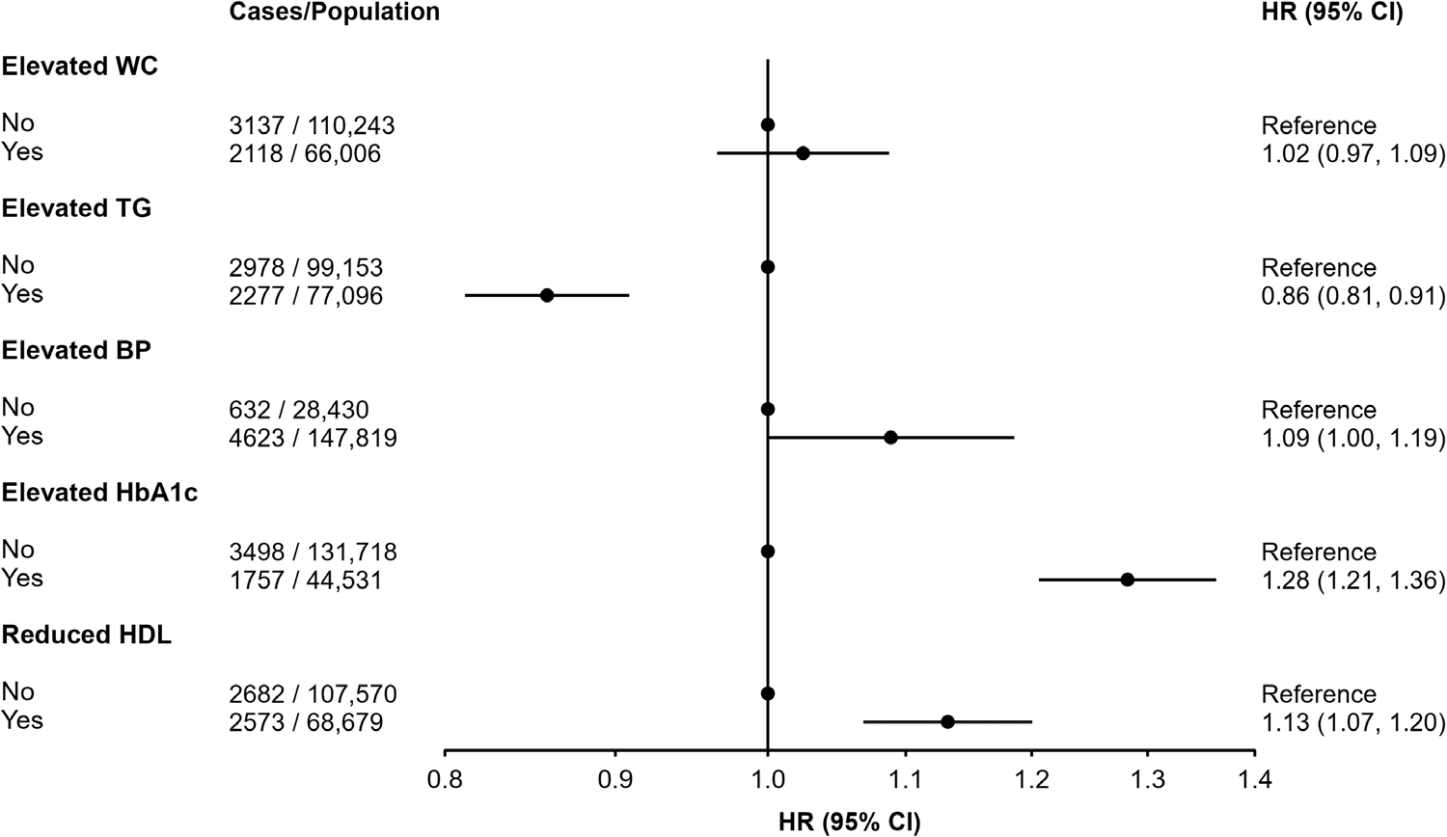
Cox proportional‐hazards model investigating the association between individual metabolic syndrome (MetS) components and incident dementia. HR = hazard ratio, CI = confidence interval, Ref. = reference group, WC = waist circumference, TG = triglycerides, BP = blood pressure, HbA1c = glycated hemoglobin A1c, HDL = high‐density lipoprotein. All individual components entered into one model. Model adjusted for age, sex, ethnicity, Townsend deprivation index, education, household income, smoking status, alcohol intake, physical activity, and APOE ε4 carrier status.

In analyses investigating individual MetS components, elevated HbA1c (HR: 1.28, 95% CI: 1.21, 1.36), reduced HDL cholesterol (HR: 1.13, 95% CI: 1.07, 1.20), and elevated blood pressure (HR: 1.09, 95% CI: 1.00, 1.19) were all associated with an increased risk of dementia (Figure 2). Conversely, elevated triglycerides were associated with a lower risk (HR: 0.86, 95% CI: 0.81, 1.91), while there was no association between elevated waist circumference and incident dementia. The findings were similar when assigning the use of lipid‐modifying medications to the elevated triglycerides group instead of the reduced HDL‐cholesterol group (elevated triglycerides HR: 0.80, 95% CI: 0.74, 0.86; reduced HDL cholesterol: HR: 1.16, 95% CI: 1.07, 1.25). Associations also remained similar when restricting the analysis to participants with >5 to 10 years and >10 years of follow‐up (Supplemental File 14).

**FIGURE 3 alz13439-fig-0003:**
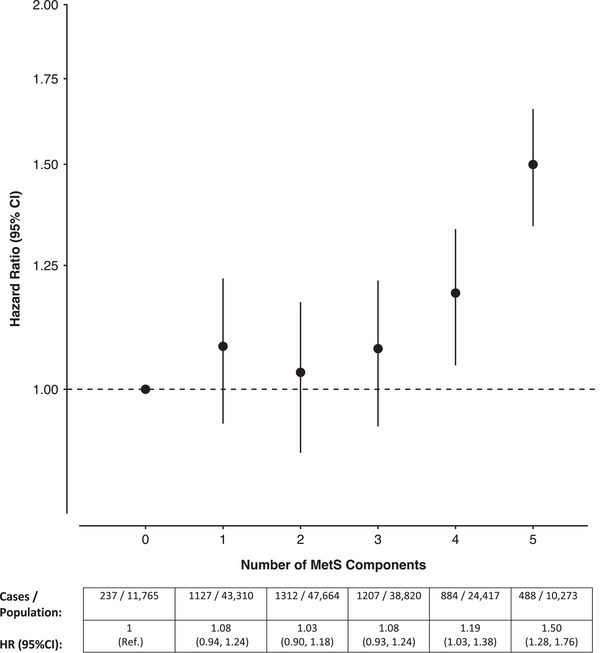
Cox proportional‐hazards model investigating the association between the number of metabolic syndrome (MetS) components present and incident dementia. HR = hazard ratio, CI = confidence interval, Ref. = reference group. Model adjusted for age, sex, ethnicity, Townsend deprivation index, education, household income, smoking status, alcohol intake, physical activity, and APOE‐ε4 carrier status.

Compared to participants with no MetS components, only the presence of four (HR: 1.19, 95% CI: 1.03, 1.38) or five (HR: 1.50, 95% CI: 1.28, 1.76) components was significantly associated with an increased risk of dementia (Figure 3). The findings generally remained similar regardless of the different combination of four MetS components present (Supplemental File 15), as well as when restricting the analysis to >5 to 10 years and >10 years of follow‐up (Supplemental File 16).

## 4 DISCUSSION

In this population‐based cohort of more than 175,000 individuals aged ≥60 years, MetS was associated with a 12% increased risk of dementia over 15 years. Associations remained similar when restricting the analysis to dementia cases diagnosed after longer follow‐up periods. Stronger associations were observed in those with four or five MetS components (regardless of what the components were) and in those with a low genetic risk for dementia based on *APOE*‐ε4 carrier status.

Our findings are consistent with previous longitudinal studies from Europe and Asia which found that MetS was associated with a statistically significant elevated risk of all‐cause dementia,^6^, ^9^ Alzheimer's disease,^6^, ^48^ and vascular dementia.^6^, ^13^, ^14^, ^48^ To our knowledge, only two comparable studies found a significant positive association with incident all‐cause dementia. A record linkage study in South Korea of over four million individuals aged ≥40 years reported a 12% increased risk of all‐cause dementia over a mean follow‐up of 4.9 years,^6^ while a Taiwanese study reported that worsened MetS (ie, those who did not have MetS at the first time point, but developed it at the second time point over a 5‐year period) was associated with a twofold increased risk of all‐cause dementia in those aged ≥65 years over a 10‐year period.^9^ In contrast, other studies have reported no association between MetS and risk of dementia and its subtypes.^7^, ^8^, ^10^, ^11^, ^12^, ^13^, ^14^ These discrepant findings may be attributed to reverse causation bias resulting from short follow‐up periods in the majority of studies (i.e., <5 years). This phenomenon was documented previously; for example, Floud et al. observed that low body mass index (BMI) was associated with an increased dementia risk during the first decade of follow‐up. However, this association considerably weakened and approached null after 15 years, indicating that these findings were substantially distorted by the effects of reverse causality.^49^ In our study, the long follow‐up duration (up to 15 years) makes our results less susceptible to reverse causation bias. Specifically, the finding that there was no association during the early years of follow‐up suggests that reverse causation is unlikely to be a major issue. If reverse causation was a major source of bias, then it is likely that the observed associations would weaken over longer follow‐up periods.[Fig alz13439-fig-0002], [Fig alz13439-fig-0003]

Our results also show a greater relative risk of dementia among *APOE*‐ε4 non‐carriers with MetS. These findings are consistent with a study that found MetS to be associated with greater cognitive decline in non‐carriers.^50^ A previous study found that ε4 non‐carriers with dementia had reduced insulin sensitivity – as reflected by lower insulin‐mediated glucose disposal rates – which may make these individuals more vulnerable to the adverse effects of hyperinsulinemia inherent in MetS.^51^

Some previous studies showed a higher risk of mortality among *APOE*‐ε4 carriers,^52^, ^53^, ^54^, ^55^ and it is therefore possible that this “competing risk” may contribute to the observed lack of an association between MetS and dementia in this group (due to carriers dying before dementia onset). However, in our sample, *APOE*‐ε4 status was strongly associated with dementia risk, and our observation of a higher relative risk in ε4 non‐carriers could be due to the greater absolute background risk of dementia among ε4 carriers. In turn, this could have led to the attenuated relative risk of dementia seen in ε4 carriers. This is supported by our finding that the absolute risk differences between those with and without MetS were greater among APOE‐ε4 carriers (1.4%) versus in non‐carriers (0.9%).

In our study, elevated HbA1c, elevated blood pressure, and reduced HDL cholesterol were associated with an increased risk of dementia, which is consistent with previous research.^56^, ^57^, ^58^ Moreover, our finding of a lack of an association between elevated waist circumference and dementia in participants aged ≥60 at baseline is consistent with previous work showing this MetS component was linked to a higher risk of late‐onset dementia when measured at age 50 years, but not after 60.^59^ Conversely, we observed an inverse association between elevated triglycerides and dementia, which also remained similar after assigning lipid‐modifying medication use to this group. Previous studies have shown elevated triglycerides to be associated with an increased dementia risk at mid‐life but a reduced risk in late life, indicating a differential impact of triglycerides across the lifespan.^60^, ^61^ In contrast, we observed an inverse association between elevated triglycerides and dementia, which may be due to reverse causation, as previous evidence indicates that dementia may lead to changes in metabolism and diet, which ultimately results in lower triglyceride levels among those affected.^62^, ^63^ However, supplementary analyses revealed that this result remained consistent across different follow‐up durations, so reverse causation is unlikely to have materially affected this association. Another explanation for this finding could be attributed to more aggressive treatment among individuals with elevated triglycerides, which could have ultimately reduced their dementia risk. Therefore, those presenting with elevated triglycerides (and deemed to be at high risk for dementia) may now have low levels due to aggressive treatment, leading to the misleading conclusion that low triglycerides are linked to a greater dementia risk. Nevertheless, we cannot rule out the possibility that this relationship may reflect a true biological association, and further research exploring the underlying mechanisms is warranted.

Additionally, previous research showed an increased risk of dementia with an increasing number of MetS components, starting with a notably elevated risk evident among those with just one component.^5^, ^64^ In contrast, our findings show that risk is only significantly elevated among those with four or five MetS components. One of the assumptions often criticized is that each possible MetS grouping contributes equally and uniformly to the risk of developing dementia and other conditions.^65^ However, our finding suggests that there is a subset of those with MetS – specifically, those with four or five components – who could significantly benefit from early treatment and prevention strategies aimed at reducing their dementia risk.

The mechanisms underlying the association between MetS and dementia remain unclear. Individual MetS components have been linked to an increased dementia risk, and the strength of these associations varies depending on whether these components were present at mid‐life versus late life.^59^, ^66^, ^67^, ^68^ Given these differential associations observed across the life course, it is important to understand whether they are driven by MetS or specific components. In MetS, the different components share universal mechanisms that could result in cognitive dysfunction and dementia through the involvement of both vascular injury and neurodegeneration. Hence, the pathogenesis behind this relationship could be multifactorial, with individual MetS components contributing independently and synergistically to dementia risk; this may be via insulin resistance, vascular endothelial damage, and oxidative stress combined with low‐grade inflammation – all of which are implicated in MetS development – which may lead to cognitive dysfunction and dementia.^69^, ^70^

To date, there is limited evidence on the potential impact of varying combinations of MetS components on dementia risk.^71^ In our study, we found that only some MetS combinations were associated with dementia risk. Notably, the combination of reduced HDL cholesterol, elevated blood pressure, and elevated triglycerides was associated with a significantly reduced risk of dementia. Taken together, these results suggest that dementia risk may vary substantially according to which MetS components are present, further highlighting the importance of investigating the contribution of different MetS combinations in future studies.

The current study has several strengths. These include using a large study population with extensive phenotypic and genotypic data collection and a long follow‐up period, which enabled us to robustly investigate the association between MetS and dementia.^21^ The present study also has several limitations. First, we used hospital inpatient admissions and death records to ascertain incident dementia cases, which likely underestimated the number of cases captured from other sources, such as primary care or memory clinics.^35^, ^72^ However, previous work demonstrated high validity for the methods used to ascertain dementia in our study.^35^, ^73^ Second, we did not investigate the association between MetS and dementia subtypes (e.g. Alzheimer's or vascular dementia) since the available medical record data have low accuracy for identifying subtypes.^35^ Third, we used HbA1c as a proxy for fasting glucose (due to the low number of fasting samples), which differs from the Harmonized Criteria for MetS; however, the American Diabetes Association recommendations support using this measure as an appropriate proxy for glucose values.^22^ Fourth, our study may be susceptible to confounding by indication, as using medications – which were previously associated with reduced dementia risk^74^ – to define specific MetS components could underestimate the true association between MetS and dementia. Finally, given the observational design of this study, residual confounding and other non‐causal explanations remain.

In the present study, we found that MetS was associated with an increased risk of dementia. The strength of the association was greatest among individuals with four or five MetS components. Given that the presence of at least three components is the established threshold for a MetS diagnosis, it is necessary to determine whether MetS is driving the associations or whether it is simply the varying combinations of individual established risk factors for dementia. This is an important next step to understand the role of MetS as a potential target for dementia prevention.

## CONFLICTS OF INTEREST STATEMENT

The authors declare no conflicts of interest.

## CONSENT STATEMENT

All participants provided electronically signed consent for their data to be used in health‐related research. UK Biobank received ethical approval from the NHS North West Centre for Research Ethics Committee (Ref: 11/NW/0382).

## Supporting information

ICMJE Disclosure Form

Supporting Information

## Data Availability

The UK Biobank Resource holds the data used in this article. Data can be accessed by application to the UK Biobank (www.ukbiobank.ac.uk/register‐apply).

## References

[1] Alberti KG , Eckel RH , Grundy SM , et al. Harmonizing the metabolic syndrome: a joint interim statement of the international diabetes federation task force on epidemiology and prevention; national heart, lung, and blood institute; American heart association; world heart federation; international atherosclerosis society; and international association for the study of obesity. Circulation. 2009;120:1640‐1645. 19805654 10.1161/CIRCULATIONAHA.109.192644

[2] AH FEGWM . Increasing prevalence of the metabolic syndrome among US adults. Diabetes Care. 2004;27:9. 15451914 10.2337/diacare.27.10.2444

[3] Wong MC , Huang J , Pang TW , et al. Tu1007 worldwide incidence and prevalence of metabolic syndrome: a systematic review and meta‐analysis of 14.6 million individuals. Gastroenterology. 2020;158:S‐1003.

[4] Atti AR , Valente S , Iodice A , et al. Metabolic syndrome, mild cognitive impairment, and dementia: a meta‐analysis of longitudinal studies. Am J Geriatr Psychiatry. 2019;27:625‐637. 30917904 10.1016/j.jagp.2019.01.214

[5] Machado‐Fragua MD , Fayosse A , Yerramalla MS , et al. Association of metabolic syndrome with incident dementia: role of number and age at measurement of components in a 28‐year follow‐up of the Whitehall II cohort study. Diabetes Care. 2022;45:2127‐2135. 35819815 10.2337/dc22-0206 PMC9472484

[6] Lee JE , Shin DW , Han K , et al. Changes in metabolic syndrome status and risk of dementia. J Clin Med. 2020;9:122. 31906539 10.3390/jcm9010122 PMC7019689

[7] Creavin ST , Gallacher J , Bayer A , Fish M , Ebrahim S , Ben‐Shlomo Y . Metabolic syndrome, diabetes, poor cognition, and dementia in the Caerphilly prospective study. J Alzheimer's Dis. 2012;28:931‐939. 22133761 10.3233/JAD-2011-111550

[8] Exalto LG , Van Der Flier WM , Van Boheemen CJ , et al. The metabolic syndrome in a memory clinic population: relation with clinical profile and prognosis. J Neurol Sci. 2015;351:18‐23. 25748296 10.1016/j.jns.2015.02.004

[9] Fan YC , Chou CC , You SL , Sun CA , Chen CJ , Bai CH . Impact of worsened metabolic syndrome on the risk of dementia: a nationwide cohort study. J Am Heart Assoc. 2017;6:e004749. 28899896 10.1161/JAHA.116.004749 PMC5634246

[10] Forti P , Pisacane N , Rietti E , et al. Metabolic syndrome and risk of dementia in older adults. J Am Geriatr Soc. 2010;58:487‐492. 20398117 10.1111/j.1532-5415.2010.02731.x

[11] Muller M , Tang M‐X , Schupf N , Manly JJ , Mayeux R , Luchsinger JA . Metabolic syndrome and dementia risk in a multiethnic elderly cohort. Dement Geriatr Cogn Disord. 2007;24:185‐192. 17641531 10.1159/000105927 PMC2268640

[12] Ng TP , Feng L , Nyunt MSZ , et al. Metabolic syndrome and the risk of mild cognitive impairment and progression to dementia: follow‐up of the Singapore longitudinal ageing study cohort. JAMA Neurol. 2016;73:456‐463. 26926205 10.1001/jamaneurol.2015.4899

[13] Raffaitin C , Gin H , Empana J‐P , et al. Metabolic syndrome and risk for incident Alzheimer's disease or vascular dementia: the Three‐City Study. Diabetes Care. 2009;32:169‐174. 18945929 10.2337/dc08-0272 PMC2606808

[14] Solfrizzi V , Scafato E , Capurso C , et al. Metabolic syndrome and the risk of vascular dementia: the Italian Longitudinal Study on Ageing. J Neurol Neurosurg Psychiatry. 2010;81:433‐440. 19965842 10.1136/jnnp.2009.181743

[15] Feng L , Chong MS , Lim WS , et al. Metabolic syndrome and amnestic mild cognitive impairment: singapore Longitudinal Ageing Study‐2 findings. J Alzheimer's Dis. 2013;34:649‐657. 23246920 10.3233/JAD-121885

[16] Jones NS , Rebeck GW . The synergistic effects of APOE genotype and obesity on Alzheimer's disease risk. Int J Mol Sci. 2018;20:63. 30586872 10.3390/ijms20010063 PMC6337558

[17] Kivipelto M , Helkala E‐L , Laakso MP , et al. Apolipoprotein E ε4 allele, elevated midlife total cholesterol level, and high midlife systolic blood pressure are independent risk factors for late‐life Alzheimer disease. Ann Intern Med. 2002;137:149‐155. 12160362 10.7326/0003-4819-137-3-200208060-00006

[18] Lai C‐L , Liou L‐M , Liu C‐K , Yang Y‐H , Lin R‐T . Effects of metabolic syndrome, apolipoprotein E, and CYP46 on cognition among Taiwanese Chinese. Kaohsiung J Med Sci. 2014;30:343‐349. 24924840 10.1016/j.kjms.2014.03.005 PMC11916575

[19] Peila R , Rodriguez BL , Launer LJ . Type 2 diabetes, APOE gene, and the risk for dementia and related pathologies: the Honolulu‐Asia Aging Study. Diabetes. 2002;51:1256‐1262. 11916953 10.2337/diabetes.51.4.1256

[20] Livingston G , Huntley J , Sommerlad A , et al. Dementia prevention, intervention, and care: 2020 report of the Lancet Commission. Lancet North Am Ed. 2020;396:413‐446. 10.1016/S0140-6736(20)30367-6 PMC7392084 32738937

[21] Sudlow C , Gallacher J , Allen N , et al. UK biobank: an open access resource for identifying the causes of a wide range of complex diseases of middle and old age. PLoS Med. 2015;12:e1001779. 25826379 10.1371/journal.pmed.1001779 PMC4380465

[22] Association AD . Standards of medical care in diabetes—2010. Diabetes Care. 2010;33:S11‐S61. 20042772 10.2337/dc10-S011 PMC2797382

[23] Ascaso J , Gonzalez Santos P , Hernandez Mijares A , et al. Management of dyslipidemia in the metabolic syndrome: recommendations of the Spanish HDL‐Forum. Am J Cardiovasc Drugs. 2007;7:39‐58. 17355165 10.2165/00129784-200707010-00004

[24] Christensen MR , Bugge A , Malik ME , et al. Establishing post mortem criteria for the metabolic syndrome: an autopsy based cross‐sectional study. Diabetol Metab Syndr. 2018;10:1‐8. 29713389 10.1186/s13098-018-0339-0 PMC5918842

[25] Haring R , Rosvall M , Völker U , et al. A network‐based approach to visualize prevalence and progression of metabolic syndrome components. PLoS One. 2012;7:e39461. 22724019 10.1371/journal.pone.0039461 PMC3378536

[26] Isomura K , Brander G , Chang Z , et al. Metabolic and cardiovascular complications in obsessive‐compulsive disorder: a total population, sibling comparison study with long‐term follow‐up. Biol Psychiatry. 2018;84:324‐331. 29395042 10.1016/j.biopsych.2017.12.003

[27] Kero A , Madanat‐Harjuoja L , Järvelä L , Malila N , Matomäki J , Lähteenmäki P . Health conditions associated with metabolic syndrome after cancer at a young age: a nationwide register‐based study. Cancer Epidemiol. 2016;41:42‐49. 26816350 10.1016/j.canep.2016.01.009

[28] Perini W , Kunst A , Snijder M , Peters R , van Valkengoed I . Ethnic differences in metabolic cardiovascular risk among normal weight individuals: implications for cardiovascular risk screening. The HELIUS study. Nutr Metab Cardiovasc Dis. 2019;29:15‐22. 30467070 10.1016/j.numecd.2018.09.004

[29] Pitchika A , Markus MRP , Schipf S , et al. Longitudinal association of Apolipoprotein E polymorphism with lipid profile, type 2 diabetes and metabolic syndrome: results from a 15 year follow‐up study. Diabetes Res Clin Pract. 2022;185:109778. 35167921 10.1016/j.diabres.2022.109778

[30] Révész D , Milaneschi Y , Verhoeven JE , Lin J , Penninx BW . Longitudinal associations between metabolic syndrome components and telomere shortening. J Clin Endocrinol Metab. 2015;100:3050‐3059. 26133009 10.1210/JC.2015-1995

[31] Runge K , van Zon SK , Bültmann U , Henkens K . Metabolic syndrome incidence in an aging workforce: occupational differences and the role of health behaviors. SSM‐Popul health. 2021;15:100881. 34401460 10.1016/j.ssmph.2021.100881 PMC8350497

[32] Suhre K , Stephan N , Zaghlool S , et al. Matching drug metabolites from non‐targeted metabolomics to self‐reported medication in the Qatar biobank study. Metabolites. 2022;12:249. 35323692 10.3390/metabo12030249 PMC8948833

[33] van Reedt Dortland AK , Giltay EJ , Van Veen T , Zitman FG , Penninx BW . Metabolic syndrome abnormalities are associated with severity of anxiety and depression and with tricyclic antidepressant use. Acta Psychiatr Scand. 2010;122:30‐39. 20456284 10.1111/j.1600-0447.2010.01565.x

[34] van Zon SK , Amick III BC , de Jong T , Brouwer S , Bültmann U . Occupational distribution of metabolic syndrome prevalence and incidence differs by sex and is not explained by age and health behavior: results from 75 000 Dutch workers from 40 occupational groups. BMJ Open Diabetes Res Care. 2020;8:e001436. 10.1136/bmjdrc-2020-001436 PMC7342434 32636219

[35] Wilkinson T , Schnier C , Bush K , et al. Identifying dementia outcomes in UK Biobank: a validation study of primary care, hospital admissions and mortality data. Eur J Epidemiol. 2019;34:557‐565. 30806901 10.1007/s10654-019-00499-1 PMC6497624

[36] Kaur J . Assessment and screening of the risk factors in metabolic syndrome. Medi Sci. 2014;2:140‐152.

[37] Townsend P , Phillimore P , Beattie A , Health and deprivation: inequality and the North: Routledge; 1988.

[38] Craig CL , Marshall AL , Sjöström M , et al. International physical activity questionnaire: 12‐country reliability and validity. Med Sci Sports Exerc. 2003;35:1381‐1395. 12900694 10.1249/01.MSS.0000078924.61453.FB

[39] Bycroft C , Freeman C , Petkova D , et al. The UK Biobank resource with deep phenotyping and genomic data. Nature. 2018;562:203‐209. 30305743 10.1038/s41586-018-0579-z PMC6786975

[40] Ebenau JL , van der Lee SJ , Hulsman M , et al. Risk of dementia in APOE ε4 carriers is mitigated by a polygenic risk score. Alzheimer's Dement: Diagn Assess Dis Monit. 2021;13:e12229. 10.1002/dad2.12229 PMC8438688 34541285

[41] De Rojas I , Moreno‐Grau S , Tesi N , et al. Common variants in Alzheimer's disease and risk stratification by polygenic risk scores. Nat Commun. 2021;12:1‐16. 34099642 10.1038/s41467-021-22491-8 PMC8184987

[42] Kunkle BW , Grenier‐Boley B , Sims R , et al. Genetic meta‐analysis of diagnosed Alzheimer's disease identifies new risk loci and implicates Aβ, tau, immunity and lipid processing. Nat Genet. 2019;51:414‐430. 30820047 10.1038/s41588-019-0358-2 PMC6463297

[43] Sims R , Van Der Lee SJ , Naj AC , et al. Rare coding variants in PLCG2, ABI3, and TREM2 implicate microglial‐mediated innate immunity in Alzheimer's disease. Nat Genet. 2017;49:1373‐1384. 28714976 10.1038/ng.3916 PMC5669039

[44] Chang CC , Chow CC , Tellier LC , Vattikuti S , Purcell SM , Lee JJ . Second‐generation PLINK: rising to the challenge of larger and richer datasets. Gigascience. 2015;4:s13742‐015‐0047‐8. 10.1186/s13742-015-0047-8 PMC4342193 25722852

[45] Arnold M , Nho K , Kueider‐Paisley A , et al. Sex and APOE ε4 genotype modify the Alzheimer's disease serum metabolome. Nat Commun. 2020;11:1‐12. 32123170 10.1038/s41467-020-14959-w PMC7052223

[46] Mielke MM , Vemuri P , Rocca WA . Clinical epidemiology of Alzheimer's disease: assessing sex and gender differences. Clin Epidemiol. 2014;6:37. 24470773 10.2147/CLEP.S37929 PMC3891487

[47] Expert Panel on Detection E . Executive summary of the third report of the National Cholesterol Education Program (NCEP) expert panel on detection, evaluation, and treatment of high blood cholesterol in adults (Adult Treatment Panel III). JAMA. 2001;285:2486‐2497. 11368702 10.1001/jama.285.19.2486

[48] Yoo H , Kim H , Koh I , Lee K , Ok J . Effect of metabolic syndrome on the incidence of dementia based on national insurance data in Korea. Metab Syndr Relat Disord. 2022;20:29‐35. 34756135 10.1089/met.2021.0046

[49] Floud S , Simpson RF , Balkwill A , et al. Body mass index, diet, physical inactivity, and the incidence of dementia in 1 million UK women. Neurology. 2020;94:e123. 31852815 10.1212/WNL.0000000000008779 PMC6988985

[50] Bangen KJ , Armstrong NM , Au R , Gross AL . Metabolic syndrome and cognitive trajectories in the framingham offspring study. J Alzheimer's Dis. 2019;71:931‐943. 31450495 10.3233/JAD-190261 PMC12854274

[51] Craft S , Asthana S , Schellenberg G , et al. Insulin metabolism in Alzheimer's disease differs according to apolipoprotein E genotype and gender. Neuroendocrinology. 1999;70:146‐152. 10461029 10.1159/000054469

[52] Jacobsen R , Martinussen T , Christiansen L , et al. Increased effect of the ApoE gene on survival at advanced age in healthy and long‐lived Danes: two nationwide cohort studies. Aging Cell. 2010;9:1004‐1009. 20849521 10.1111/j.1474-9726.2010.00626.x PMC2988163

[53] Joshi PK , Fischer K , Schraut KE , Campbell H , Esko T , Wilson JF . Variants near CHRNA3/5 and APOE have age‐and sex‐related effects on human lifespan. Nat Commun. 2016;7:11174. 27029810 10.1038/ncomms11174 PMC5438072

[54] Rajan KB , Barnes LL , Wilson RS , et al. Racial differences in the association between apolipoprotein E risk alleles and overall and total cardiovascular mortality over 18 years. J Am Geriatr Soc. 2017;65:2425‐2430. 28898389 10.1111/jgs.15059 PMC6201232

[55] Wolters FJ , Yang Q , Biggs ML , et al. The impact of APOE genotype on survival: results of 38,537 participants from six population‐based cohorts (E2‐CHARGE). PLoS One. 2019;14:e0219668. 31356640 10.1371/journal.pone.0219668 PMC6663005

[56] Ou YN , Tan CC , Shen XN , et al. Blood pressure and risks of cognitive impairment and dementia: a systematic review and meta‐analysis of 209 prospective studies. Hypertension. 2020;76:217‐225. 32450739 10.1161/HYPERTENSIONAHA.120.14993

[57] Xue M , Xu W , Ou YN , et al. Diabetes mellitus and risks of cognitive impairment and dementia: a systematic review and meta‐analysis of 144 prospective studies. Ageing Res Rev. 2019;55:100944. 31430566 10.1016/j.arr.2019.100944

[58] Zhang X , Tian Q , Liu D , et al. Causal association of circulating cholesterol levels with dementia: a mendelian randomization meta‐analysis. Transl Psychiatry. 2020;10:145. 32398686 10.1038/s41398-020-0822-x PMC7217910

[59] Singh‐Manoux A , Dugravot A , Shipley M , et al. Obesity trajectories and risk of dementia: 28 years of follow‐up in the Whitehall II Study. Alzheimer's Dis. 2018;14:178‐186. 10.1016/j.jalz.2017.06.2637 PMC5805839 28943197

[60] Anstey KJ , Ashby‐Mitchell K , Peters R . Updating the evidence on the association between serum cholesterol and risk of late‐life dementia: review and meta‐analysis. J Alzheimer's Dis. 2017;56:215‐228. 27911314 10.3233/JAD-160826 PMC5240556

[61] Gong J , Harris K , Peters SA , Woodward M . Serum lipid traits and the risk of dementia: a cohort study of 254,575 women and 214,891 men in the UK Biobank. Eclinicalmedicine. 2022;54:101695. 36247924 10.1016/j.eclinm.2022.101695 PMC9561731

[62] Dimopoulos N , Piperi C , Salonicioti A , et al. Characterization of the lipid profile in dementia and depression in the elderly. J Geriatr Psychiatry Neurol. 2007;20:138‐144. 17712096 10.1177/0891988707301867

[63] Lepara O , Valjevac A , Alajbegović A , Zaćiragić A , Nakaš‐Ićindić E . Decreased serum lipids in patients with probable Alzheimer's disease. Bosn J Basic Med Sci. 2009;9:215. 19754476 10.17305/bjbms.2009.2809 PMC5632505

[64] Cho Y , Han K , Kim DH , et al. Cumulative exposure to metabolic syndrome components and the risk of dementia: a nationwide population‐based study. Endocrinol Metab. 2021;36:424‐435. 10.3803/EnM.2020.935 PMC8090478 33849249

[65] Moebus S , Balijepalli C , Lösch C , et al. Age‐and sex‐specific prevalence and ten‐year risk for cardiovascular disease of all 16 risk factor combinations of the metabolic syndrome‐A cross‐sectional study. Cardiovasc Diabetol. 2010;9:1‐12. 20696055 10.1186/1475-2840-9-34 PMC2929217

[66] Kivimäki M , Luukkonen R , Batty GD , et al. Body mass index and risk of dementia: analysis of individual‐level data from 1.3 million individuals. Alzheimer's Dis. 2018;14:601‐609. 10.1016/j.jalz.2017.09.016 PMC5948099 29169013

[67] Walker KA , Sharrett AR , Wu A , et al. Association of midlife to late‐life blood pressure patterns with incident dementia. JAMA. 2019;322:535‐545. 31408138 10.1001/jama.2019.10575 PMC6692677

[68] Kivipelto M , Helkala EL , Laakso MP , et al. Midlife vascular risk factors and Alzheimer's disease in later life: longitudinal, population based study. BMJ. 2001;322:1447‐1451. 11408299 10.1136/bmj.322.7300.1447 PMC32306

[69] Arshad N , Lin TS , Yahaya MF . Metabolic syndrome and its effect on the brain: possible mechanism. CNS Neurol Disord Drug Targets. 2018;17:595‐603. 30047340 10.2174/1871527317666180724143258

[70] Borshchev YY , Uspensky YP , Galagudza MM . Pathogenetic pathways of cognitive dysfunction and dementia in metabolic syndrome. Life Sci. 2019;237:116932. 31606384 10.1016/j.lfs.2019.116932

[71] Anstey KJ , Peters R , Zheng L , et al. Future directions for dementia risk reduction and prevention research: an international research network on dementia prevention consensus. J Alzheimer's Dis. 2020;78:3‐12. 32925063 10.3233/JAD-200674 PMC7609069

[72] Wilkinson T , Ly A , Schnier C , et al. Identifying dementia cases with routinely collected health data: a systematic review. Alzheimer's Dis. 2018;14:1038‐1051. 10.1016/j.jalz.2018.02.016 PMC6105076 29621480

[73] Reiman EM , Langbaum JB , Tariot PN . Alzheimer's prevention initiative: a proposal to evaluate presymptomatic treatments as quickly as possible. Biomark Med. 2010;4:3‐14. 20383319 10.2217/bmm.09.91 PMC2850446

[74] Larsson SC , Markus HS . Does treating vascular risk factors prevent dementia and Alzheimer's disease? A systematic review and meta‐analysis. J Alzheimer's Dis. 2018;64:657‐668. 29914039 10.3233/JAD-180288

