# Supplementary material for: Association between metabolic syndrome and risk of incident dementia in UK Biobank
Source: Alzheimers Dement. 2023 Sep 7;20(1):447–58. doi: 10.1002/alz.13439 (PMC10916994; doi:10.1002/alz.13439)
Supplement: Supplementary file 1 — ICMJE Disclosure Form [file ALZ-20-447-s002.docx]

**Supplemental File 1: Cohort Flow Diagram**

All participants in the UK Biobank study (n= 502,414)

Multiple Imputation Analysis

n=217,297

Excluded:

♦  Participants aged <60 years (n=284,951)

♦  Participants with prevalent dementia (n=166)

Complete Case Analysis

n=176,249

Participants with available data on all five metabolic syndrome components

n=176,249 (81.1%)

UK Biobank participants aged ≥60 years without a prior history of dementia (n=217,297)

Participants with ≥1 missing value among any of the five metabolic syndrome components

n=41,048 (18.9%)

## Analysis

**Supplemental File 2: Variables and medication codes used to define MetS in the UKB cohort**

| **Metabolic syndrome component** | **Harmonized criteria (2009)**  ***Three or more of the following:*** | **UKB data field ID** | **Field code description** |
| --- | --- | --- | --- |
| **Elevated waist circumference** | Elevated waist circumference: ≥ 102 cm in males and ≥ 88 cm in females | 48 | Waist circumference |
| **Elevated triglycerides** | ≥ 150 mg/dL (1.7 mmol/L) | 30870 | Triglycerides Blood Biochemistry; |
| **Elevated blood pressure** | Systolic: ≥130 mmHg and/or Diastolic ≥85 mmHg*, or antihypertensive medication use  *In UKB, BP was measured twice by trained nurses after participants had been at rest for at least 5 minutes. Individual systolic and diastolic BP measurements were averaged within a visit. Automated BP readings were the preferred source of data. However, if this was unavailable, manual readings were used | 4080, 4079;  93,94 | Systolic BP - automated reading, Diastolic BP - automated reading;  Systolic BP - manual reading, Diastolic BP - manual reading |
|  |  | 20003 | ATC Codes starting with: C02, C03, C07, C08, C09 |
| **Elevated blood glucose** | ≥100 mg/dL (≥5.6 mmol/L) †, or drug treatment for elevated glucose level  †HbA1c used as a proxy indicator, with cut-offs based on the recommendations of the American Diabetes Association: HbA1c ≥5.7% | 30750 | Glycated haemoglobin (hbA1C) |
|  |  | 20003 | ATC Codes starting with: A10 |
| **Reduced HDL cholesterol** | Males: <40 mg/dL (1.0 mmol/L); Females: <50 mg/dL (1.3 mmol/L), or lipid-modifying medications | 30760 | HDL cholesterol |
|  |  | 20003 | ATC Codes starting with: C10 |

*UKB = UK Biobank, ATC = Anatomical Therapeutic Chemical, HDL = High-density lipoprotein, BP = Blood pressure, HbA1c = Glycated haemoglobin*

**Supplemental File 3: Detailed description of UKB medication codes (mapped to ATC codes) used to define MetS components**

| **UKB Drug Category** | **UKB Drug Name** | **UKB Code** | **ATC Code** |
| --- | --- | --- | --- |
| ***MEDICATIONS FOR REDUCED HDL CHOLESTEROL*** | | | |
| atorvastatin | Atorvastatin | 1141146234 | C10AA05 |
| lipitor 10mg tablet | Atorvastatin | 1141146138 | C10AA05 |
| rosuvastatin | Rosuvastatin | 1141192410 | C10AA07 |
| crestor 10mg tablet | Rosuvastatin | 1141192414 | C10AA07 |
| simvastatin | Simvastatin | 1140861958 | C10AA01 |
| simvador 10mg tablet | Simvastatin | 1141188146 | C10AA01 |
| zocor 10mg tablet | Simvastatin | 1140881748 | C10AA01 |
| pravastatin | Pravastatin | 1140888648 | C10AA03 |
| lipostat 10mg tablet | Pravastatin | 1140861970 | C10AA03 |
| ezetimibe | Ezetimibe | 1141192736 | C10AX09 |
| ezetrol 10mg tablet | Ezetimibe | 1141192740 | C10AX09 |
| fenofibrate | Fenofibrate | 1140861954 | C10AB05 |
| lipantil micro 67mg capsule | Fenofibrate | 1141162544 | C10AB05 |
| supralip 160mg m/r tablet | Fenofibrate | 1141172214 | C10AB05 |
| fluvastatin | Fluvastatin | 1140888594 | C10AA04 |
| lescol 20mg capsule | Fluvastatin | 1140864592 | C10AA04 |
| gemfibrozil | Gemfibrozil | 1140861856 | C10AB04 |
| omacor 1g capsule | Omega-3-Acid Ethyl Esters | 1141181868 | C10AX06 |
| bezafibrate | Bezafibrate | 1140861924 | C10AB02 |
| questran 4g/sachet powder | Cholestyramine | 1140861936 | C10AC01 |
| colestyramine | Cholestyramine | 1140909780 | C10AC01 |
| ciprofibrate | Ciprofibrate | 1140862026 | C10AB08 |
| bezalip-mono 400mg m/r tablet | Bezafibrate | 1140861928 | C10AB02 |
| bezalip 200mg tablet | Bezafibrate | 1140861926 | C10AB02 |
| niacin | Niacin | 1140910670 | C04AC01 \|C10AD02 |
| niaspan 500mg m/r tablet | Niacin | 1141188546 | C10AD02 |
| nicotinic acid product | Niacin | 1140861868 | C04AC01 \|C10AD02 |
| cholestyramine | Cholestyramine | 1140865576 | C10AC01 |
| fibrazate xl 400mg m/r tablet | Bezafibrate | 1141201306 | C10AB02 |
| colestyramine product | Cholestyramine | 1141180734 | C10AC01 |
| bezafibrate product | Bezafibrate | 1141157260 | C10AB02 |
| modalim 100mg tablet | Ciprofibrate | 1140862028 | C10AB08 |
| zimbacol xl 400mg m/r tablet | Bezafibrate | 1141171548 | C10AB02 |
| colestipol | Colestipol | 1140888590 | C10AC02 |
| colestid 5g/sachet granules | Colestipol | 1140861848 | C10AC02 |
| acipimox | Acipimox | 1140861892 | C10AD06 |
| colestyramine+aspartame 4g/sachet powder | Cholestyramine | 1141180722 | C10AC01 |
| ***MEDICATIONS FOR ELEVATED BLOOD PRESSURE*** | | | |
| hydralazine | Hydralazine | 1140888686 | C02DB02 |
| moxonidine | Moxonidine | 1140928284 | C02AC05 |
| physiotens 200micrograms tablet | Moxonidine | 1140928290 | C02AC05 |
| minoxidil | Minoxidil | 1140860532 | C02DC01 \|D11AX01 |
| doxazosin | Doxazosin | 1140879778 | C02CA04 |
| methyldopa | Methyldopa | 1140860470 | C02AB |
| cardura 1mg tablet | Doxazosin | 1140860690 | C02CA04 |
| doxadura 1mg tablet | Doxazosin | 1141194372 | C02CA04 |
| clonidine | Clonidine | 1140883468 | C02AC01 \|N02CX02 \|S01EA04 |
| indoramin | Indoramin | 1140879782 | C02CA02 |
| prazosin | Prazosin | 1140879794 | C02CA01 |
| hypovase 500mcg tablet | Prazosin | 1140860580 | C02CA01 |
| indapamide | Indapamide | 1140866078 | C03BA11 |
| natrilix sr 1.5mg m/r tablet | Indapamide | 1141146378 | C03BA11 |
| hydrochlorothiazide | Hydrochlorothiazide | 1140866162 | C03AA03 |
| furosemide | Furosemide | 1140909708 | C03CA01 |
| frusemide | Furosemide | 1140866116 | C03CA01 |
| spironolactone | Spironolactone | 1140866236 | C03DA01 |
| spirolone 25mg tablet | Spironolactone | 1140866318 | C03DA01 |
| chlortalidone | Chlorthalidone | 1140909706 | C03BA04 |
| eplerenone | Eplerenone | 1141201244 | C03DA04 |
| hygroton 50mg tablet | Chlorthalidone | 1140866146 | C03BA04 |
| metolazone | Metolazone | 1140866092 | C03BA08 |
| bendroflumethiazide | Bendroflumethiazide | 1141194794 | C03AA01 |
| bendrofluazide | Bendroflumethiazide | 1140866122 | C03AA01 |
| co-amilofruse | Amiloride \|Furosemide | 1140923402 | C03EB01 |
| bumetanide | Bumetanide | 1140866280 | C03CA02 |
| co-amilozide | Amiloride \|Hydrochlorothiazide | 1140923276 | C03EA01 |
| amiloride | Amiloride | 1140888512 | C03DB01 |
| bendroflumethiazide+potassium 2.5mg/7.7mmol m/r tablet | Bendroflumethiazide \|Potassium | 1141194800 | C03AB01 |
| frumil tablet | Amiloride \|Furosemide | 1140866406 | C03CA01 |
| bendrofluazide+potassium 2.5mg/7.7mmol m/r tablet | Bendroflumethiazide \|Potassium | 1140866450 | C03AB01 |
| bzt - bendrofluazide | Bendroflumethiazide | 1140910442 | C03AA01 |
| dyazide tablet | Triamterene \|Hydrochlorothiazide | 1140866402 | C03EA01 |
| moduretic tablet | Amiloride \|Hydrochlorothiazide | 1140866420 | C03EA01 |
| navispare tablet | Amiloride \|Cyclopenthiazide | 1140866352 | C03EA07 |
| co-triamterzide | Triamterene \|Hydrochlorothiazide | 1140923272 | C03EA01 |
| torasemide | Torasemide | 1140888496 | C03CA04 |
| moduret 25 tablet | Amiloride \|Hydrochlorothiazide | 1140866416 | C03EA01 |
| burinex a tablet | Bumetanide | 1140866356 | C03CA02 |
| xipamide | Xipamide | 1140866108 | C03BA10 |
| cyclopenthiazide | Cyclopenthiazide | 1140866156 | C03AA07 |
| bisoprolol | Bisoprolol | 1140879760 | C07AB07 |
| cardicor 1.25mg tablet | Bisoprolol | 1141171152 | C07AB07 |
| atenolol | Atenolol | 1140866738 | C07AB03 |
| tenormin 25 tablet | Atenolol | 1140866756 | C07AB03 |
| metoprolol | Metoprolol | 1140879818 | C07AB02 |
| carvedilol | Carvedilol | 1140909368 | C07AG02 |
| propranolol | Propranolol | 1140879842 | C07AA05 |
| timolol | Timolol | 1140879866 | C07AA06 \|S01ED01 |
| half-inderal la 80mg m/r capsule | Propranolol | 1140866800 | C07AA05 |
| inderal 10mg tablet | Propranolol | 1140866804 | C07AA05 |
| bedranol 10mg tablet | Propranolol | 1140851556 | C07AA05 |
| half beta-prograne 80mg m/r capsule | Propranolol | 1140866802 | C07AA05 |
| labetalol | Labetalol | 1140879824 | C07AG01 |
| sotalol | Sotalol | 1140879854 | C07AA07 |
| beta-cardone 40mg tablet | Sotalol | 1140860304 | C07AA07 |
| co-tenidone | Chlorthalidone \|Atenolol | 1140923336 | C07CB03 |
| nebivolol | Nebivolol | 1141164276 | C07AB12 |
| bisoprolol fumarate+hydrochlorothiazide 10mg/6.25mg tablet | Bisoprolol \|Hydrochlorothiazide | 1140864950 | C07BB07 |
| celiprolol | Celiprolol | 1140879762 | C07AB08 |
| atenolol+bendroflumethiazide | Atenolol \|Bendroflumethiazide | 1141194810 | C07BB03 |
| nebilet 5mg tablet | Nebivolol | 1141164280 | C07AB12 |
| propranolol hydrochloride+bendrofluazide 80mg/2.5mg capsule | Bendroflumethiazide \|Propranolol | 1140860418 | C07BA05 |
| sotalol hydrochloride+hydrochlorothiazide 80mg/12.5mg tablet | Sotalol \|Hydrochlorothiazide | 1140860332 | C07BA07 |
| tenoret 50 tablet | Chlorthalidone \|Atenolol | 1140860324 | C07CB03 |
| carteolol | Carteolol | 1140879822 | C07AA15 \|S01ED05 |
| betaxolol | Betaxolol | 1140879758 | C07AB05 \|S01ED02 |
| atenolol+bendrofluazide | Atenolol \|Bendroflumethiazide | 1141146126 | C07BB03 |
| tenif capsule | Atenolol \|Nifedipine | 1140860358 | C07FB03 |
| metoprolol tartrate+chlorthalidone 100mg/12.5mg tablet | Metoprolol \|Chlorthalidone | 1140860308 | C07CB02 |
| tenoretic tablet | Chlorthalidone \|Atenolol | 1140860328 | C07CB03 |
| beta-adalat capsule | Atenolol \|Nifedipine | 1140860356 | C07FB03 |
| celectol 200mg tablet | Celiprolol | 1140860498 | C07AB08 |
| acebutolol | Acebutolol | 1140866724 | C07AB04 |
| oxprenolol | Oxprenolol | 1140879830 | C07AA02 |
| prindolol | Pindolol | 1140910614 | C07AA17 |
| kalten capsule | Atenolol \|Amiloride \|Hydrochlorothiazide | 1140860398 | C07DB01 |
| nadolol | Nadolol | 1140860192 | C07AA12 |
| pindolol | Pindolol | 1140860292 | C07AA03 |
| atenolol+chlortalidone | Chlorthalidone \|Atenolol | 1141180778 | C07BB03 |
| amlodipine | Amlodipine | 1140879802 | C08CA01 |
| istin 5mg tablet | Amlodipine | 1140861202 | C08CA01 |
| amlostin 5mg tablet | Amlodipine | 1141200400 | C08CA01 |
| nifedipine | Nifedipine | 1140861088 | C08CA05 |
| adalat 5mg capsule | Nifedipine | 1140861090 | C08CA05 |
| coracten sr 10mg m/r capsule | Nifedipine | 1140861120 | C08CA05 |
| adalate 10mg capsule | Nifedipine | 1140881702 | C08CA05 |
| adipine mr 10 m/r tablet | Nifedipine | 1140923572 | C08CA05 |
| fortipine la40 m/r tablet | Nifedipine | 1141145870 | C08CA05 |
| nifedipress mr 10 m/r tablet | Nifedipine | 1141157140 | C08CA05 |
| tensipine mr 10 m/r tablet | Nifedipine | 1140927940 | C08CA05 |
| verapamil | Verapamil | 1140888510 | C08DA01 |
| securon 40mg tablet | Verapamil | 1140866466 | C08DA01 |
| half securon sr 120mg m/r tablet | Verapamil | 1140866460 | C08DA01 |
| univer 120mg m/r capsule | Verapamil | 1140881692 | C08DA01 |
| vertab sr 240 m/r tablet | Verapamil | 1141169710 | C08DA01 |
| diltiazem | Diltiazem | 1140879806 | C05AE03 \|C08DB01 |
| tildiem 60mg m/r tablet | Diltiazem | 1140861128 | C08DB01 |
| adizem-60 m/r tablet | Diltiazem | 1140861138 | C08DB01 |
| adizem-xl plus m/r capsule | Diltiazem | 1140926780 | C08DB01 |
| dilzem sr 60mg long acting m/r capsule | Diltiazem | 1140861166 | C08DB01 |
| slozem 120mg m/r capsule | Diltiazem | 1140911698 | C08DB01 |
| angitil sr 90 m/r capsule | Diltiazem | 1140917428 | C08DB01 |
| viazem xl 120mg m/r capsule | Diltiazem | 1141151474 | C08DB01 |
| zemtard 120 xl m/r capsule | Diltiazem | 1141167832 | C08DB01 |
| calcicard 60mg tablet | Diltiazem | 1140851730 | C08DB01 |
| felodipine | Felodipine | 1140888646 | C08CA02 |
| cardioplen xl 5mg m/r tablet | Felodipine | 1141199858 | C08CA02 |
| vascalpha 5mg m/r tablet | Felodipine | 1141190160 | C08CA02 |
| felendil xl 5mg m/r tablet | Felodipine | 1141188836 | C08CA02 |
| plendil 2.5mg m/r tablet | Felodipine | 1140928212 | C08CA02 |
| felotens xl 5mg m/r tablet | Felodipine | 1141188152 | C08CA02 |
| neofel xl 5mg m/r tablet | Felodipine | 1141200782 | C08CA02 |
| felogen xl 5mg m/r tablet | Felodipine | 1141188576 | C08CA02 |
| cabren 2.5mg m/r tablet | Felodipine | 1141187094 | C08CA02 |
| lercanidipine | Lercanidipine | 1141153026 | C08CA13 |
| zanidip 10mg tablet | Lercanidipine | 1141153032 | C08CA13 |
| lacidipine | Lacidipine | 1140861276 | C08CA09 |
| diltiazem hcl+hydrochlorothiazide 150mg/12.5mg m/r capsule | Diltiazem \|Hydrochlorothiazide | 1140926778 | C08GA |
| nicardipine | Nicardipine | 1140879810 | C08CA04 |
| motens 2mg tablet | Lacidipine | 1140861282 | C08CA09 |
| cardene 20mg capsule | Nicardipine | 1140861176 | C08CA04 |
| valsartan | Valsartan | 1141145660 | C09CA03 |
| diovan 40mg capsule | Valsartan | 1141145668 | C09CA03 |
| losartan | Losartan | 1140916356 | C09CA01 |
| cozaar 25mg tablet | Losartan | 1141179974 | C09CA01 |
| perindopril | Perindopril | 1140888560 | C09AA04 |
| coversyl 2mg tablet | Perindopril | 1140860802 | C09AA04 |
| lisinopril | Lisinopril | 1140860696 | C09AA03 |
| zestril 2.5mg tablet | Lisinopril | 1140860714 | C09AA03 |
| irbesartan | Irbesartan | 1141152998 | C09CA04 |
| aprovel 75mg tablet | Irbesartan | 1141153006 | C09CA04 |
| enalapril | Enalapril | 1140888552 | C09AA02 |
| innovace 2.5mg tablet | Enalapril | 1140860776 | C09AA02 |
| fosinopril | Fosinopril | 1140888556 | C09AA09 |
| ramipril | Ramipril | 1140860806 | C09AA05 |
| telmisartan | Telmisartan | 1141166006 | C09CA07 |
| micardis 20mg tablet | Telmisartan | 1141172492 | C09CA07 |
| tritace 1.25mg tablet | Ramipril | 1141188408 | C09AA05 |
| lopace 2.5mg capsule | Ramipril | 1141199940 | C09AA05 |
| candesartan cilexetil | Candesartan | 1141156836 | C09CA06 |
| amias 2mg tablet | Candesartan | 1141156846 | C09CA06 |
| cilazapril | Cilazapril | 1140860882 | C09AA08 |
| olmesartan | Olmesartan | 1141193282 | C09CA08 |
| losartan potassium+hydrochlorothiazide 50mg/12.5mg tablet | Losartan \|Hydrochlorothiazide | 1141151016 | C09DA01 |
| olmetec 10mg tablet | Olmesartan | 1141193346 | C09CA08 |
| trandolapril | Trandolapril | 1140860904 | C09AA10 |
| eprosartan | Eprosartan | 1141171336 | C09CA02 |
| captopril | Captopril | 1140860750 | C09AA01 |
| quinapril | Quinapril | 1140860728 | C09AA06 |
| lisinopril+hydrochlorothiazide 10mg/12.5mg tablet | Lisinopril \|Hydrochlorothiazide | 1140864952 | C09BA03 |
| coaprovel 150mg/12.5mg tablet | Hydrochlorothiazide \|Irbesartan | 1141172686 | C09DA04 |
| enalapril maleate+hydrochlorothiazide 20mg/12.5mg tablet | Enalapril \|Hydrochlorothiazide | 1140860790 | C09BA02 |
| irbesartan+hydrochlorothiazide 150mg/12.5mg tablet | Hydrochlorothiazide \|Irbesartan | 1141172682 | C09DA04 |
| cozaar-comp 50mg/12.5mg tablet | Losartan \|Hydrochlorothiazide | 1141151018 | C09DA01 |
| perindopril+indapamide | Perindopril \|Indapamide | 1141180592 | C09BA04 |
| zestoretic 10 tablet | Lisinopril \|Hydrochlorothiazide | 1140864618 | C09BA03 |
| coversyl plus 4mg/1.25mg tablet | Perindopril \|Indapamide | 1141180598 | C09BA04 |
| co-diovan 80mg/12.5mg tablet | Valsartan \|Hydrochlorothiazide | 1141201040 | C09DA03 |
| valsartan+hydrochlorothiazide 80mg/12.5mg tablet | Valsartan \|Hydrochlorothiazide | 1141201038 | C09DA03 |
| teveten 300mg tablet | Eprosartan | 1141171344 | C09CA02 |
| micardisplus 40mg/12.5mg tablet | Telmisartan \|Hydrochlorothiazide | 1141187790 | C09DA07 |
| telmisartan+hydrochlorothiazide 40mg/12.5mg tablet | Telmisartan \|Hydrochlorothiazide | 1141187788 | C09DA07 |
| felodipine+ramipril | Ramipril \|Felodipine | 1141165470 | C09BB05 |
| imidapril hydrochloride | Imidapril | 1141164148 | C09AA16 |
| innozide tablet | Enalapril \|Hydrochlorothiazide | 1140860784 | C09BA02 |
| gopten 500micrograms capsule | Trandolapril | 1140860912 | C09AA10 |
| capozide tablet | Hydrochlorothiazide \|Captopril | 1140881714 | C09BA01 |
| capoten 12.5mg tablet | Captopril | 1140860758 | C09AA01 |
| carace 10 plus tablet | Lisinopril \|Hydrochlorothiazide | 1140864910 | C09BA03 |
| triapin mite 2.5mg/2.5mg tablet | Ramipril \|Felodipine | 1141165476 | C09BB05 |
| accupro 5mg tablet | Quinapril | 1140881706 | C09AA06 |
| ***MEDICATIONS FOR ELEVATED BLOOD GLUCOSE*** | | | |
| metformin | Metformin | 1140884600 | A10BA02 |
| glucophage 500mg tablet | Metformin | 1140874686 | A10BA02 |
| insulin product | Insulin | 1140883066 | A10A |
| gliclazide | Gliclazide | 1140874744 | A10BB09 |
| glyclizide | Gliclazide | 1140910566 | A10BB09 |
| diamicron 80mg tablet | Gliclazide | 1140874746 | A10BB09 |
| glimepiride | Glimepiride | 1141152590 | A10BB12 |
| amaryl 1mg tablet | Glimepiride | 1141156984 | A10BB12 |
| pioglitazone | Pioglitazone | 1141171646 | A10BG03 |
| actos 15mg tablet | Pioglitazone | 1141171652 | A10BG03 |
| glibenclamide | Glyburide | 1140874718 | A10BB01 |
| repaglinide | Repaglinide | 1141168660 | A10BX02 |
| rosiglitazone | Rosiglitazone | 1141177600 | A10BG02 |
| rosiglitazone 1mg / metformin 500mg tablet | Metformin \|Rosiglitazone | 1141189090 | A10BD03 |
| avandamet 1mg / 500mg tablet | Metformin \|Rosiglitazone | 1141189094 | A10BD03 |
| glipizide | Glipizide | 1140874646 | A10BB07 |
| avandia 4mg tablet | Rosiglitazone | 1141177606 | A10BG02 |
| tolbutamide | Tolbutamide | 1140874674 | A10BB03 \|V04CA01 |
| acarbose | Acarbose | 1140868902 | A10BF01 |
| nateglinide | Nateglinide | 1141173882 | A10BX03 |

**Supplemental File 4: International Classification of Disease codes used to ascertain dementia**

| **ICD-9** | **ICD-10** |
| --- | --- |
| 331.0, 290.4, 331.1, 290.2, 290.3, 291.2, 294.1, 331.2, 331.5 | F00, F00.0, F00.1, F00.2, F00.9, G30, G30.0, G30.1, G30.8, G30.9, F01, F01.0, F01.1, F01.2, F01.3, F01.8, F01.9, I67.3, F02.0, G31.0, A81.0, F02, F02.1, F02.2, F02.3, F02.4, F02.8, F03, F05.1, F10.6, G31.1, G31.8 |

*Abbreviations: ICD, International Classification of Disease*

**Supplemental File 5: Multiple imputation description**

We used the Multiple Imputation by Chained Equations (MICE) package in RStudio version 4.2.2 to impute data on missing values for individual MetS components and key covariates. Specifically, missing data were imputed for the following MetS components: waist circumference, triglycerides, blood pressure, HbA1c, and HDL cholesterol. The covariates included in the imputation model were age, sex, ethnicity, Townsend deprivation index score, education, household income, smoking status, alcohol intake, physical activity and APOE-ε4 carrier status.

The imputed MetS component values were then used to define the binary MetS variable (as per the 2009 Harmonized Criteria) in each imputed dataset. We used estimates from each dataset to produce a pooled estimate, following the rules outlined by Rubin [1-2]

[1] Rubin, D. B. (2004). Multiple imputation for nonresponse in surveys, John Wiley & Sons.

[2] White, I. R., et al. (2011). "Multiple imputation using chained equations: issues and guidance for practice." Statistics in medicine **30**(4): 377-399.

| Supplemental File 6: Baseline characteristics according to missing data on MetS components | | | | |
| --- | --- | --- | --- | --- |
| Characteristic | | **Non-Missing**  **(N=176,249)** | **Missing (N=41,048)** | **Overall (N=217,297)** |
| Age (years) | Mean (SD) | 64.1 (2.85) | 64.2 (2.85) | 64.1 (2.85) |
| Sex | Female | 92052 (52.2%) | 22560 (55.0%) | 114612 (52.7%) |
|  | Male | 84197 (47.8%) | 18488 (45.0%) | 102685 (47.3%) |
| Ethnicity | White | 170674 (96.8%) | 39043 (95.1%) | 209717 (96.5%) |
|  | Non-White | 4764 (2.7%) | 1635 (4.0%) | 6399 (2.9%) |
|  | Missing | 811 (0.5%) | 370 (0.9%) | 1181 (0.5%) |
| Education level | Primary | 47225 (26.8%) | 11150 (27.2%) | 58375 (26.9%) |
|  | Secondary | 77685 (44.1%) | 17836 (43.5%) | 95521 (44.0%) |
|  | Post-secondary non-tertiary | 18922 (10.7%) | 4294 (10.5%) | 23216 (10.7%) |
|  | Tertiary | 30021 (17.0%) | 7172 (17.5%) | 37193 (17.1%) |
|  | Missing | 2396 (1.4%) | 596 (1.5%) | 2992 (1.4%) |
| Townsend deprivation index, quintiles | 1 (least deprived) | 35508 (20.2%) | 7915 (19.3%) | 43423 (20.0%) |
|  | 2 | 35282 (20.0%) | 8141 (19.9%) | 43423 (20.0%) |
|  | 3 | 35292 (20.0%) | 8131 (19.8%) | 43423 (20.0%) |
|  | 4 | 35363 (20.1%) | 8059 (19.7%) | 43422 (20.0%) |
|  | 5 (most deprived) | 34656 (19.7%) | 8766 (21.4%) | 43422 (20.0%) |
|  | Missing | 48 (0.1%) | 36 (0.1%) | 184 (0.1%) |
| Household income (in GBP) | Less than 18,000 | 48685 (27.6%) | 11355 (27.7%) | 60040 (27.6%) |
|  | 18,000 to 30,999 | 46630 (26.5%) | 10779 (26.3%) | 57409 (26.4%) |
|  | 31,000 to 51,999 | 29936 (17.0%) | 6723 (16.4%) | 36659 (16.9%) |
|  | 52,000 to 100,000 | 14437 (8.2%) | 3228 (7.9%) | 17665 (8.1%) |
|  | Greater than 100,000 | 3409 (1.9%) | 803 (2.0%) | 4212 (1.9%) |
|  | Missing | 33152 (18.8%) | 8160 (19.9%) | 41312 (19.0%) |
| Smoking status | Never | 87285 (49.5%) | 20538 (50.0%) | 107823 (49.6%) |
|  | Previous | 73392 (41.6%) | 16709 (40.7%) | 90101 (41.5%) |
|  | Current | 14508 (8.2%) | 3384 (8.2%) | 17892 (8.2%) |
|  | Missing | 1064 (0.6%) | 417 (1.0%) | 1481 (0.7%) |
| Alcohol intake | Never | 8430 (4.8%) | 2084 (5.1%) | 10514 (4.8%) |
|  | Former drinker | 6642 (3.8%) | 1640 (4.0%) | 8282 (3.8%) |
|  | Special occasions only | 21283 (12.1%) | 5137 (12.5%) | 26420 (12.2%) |
|  | 1-3 times per month | 17401 (9.9%) | 4046 (9.9%) | 21447 (9.9%) |
|  | 1-2 times per week | 42078 (23.9%) | 9677 (23.6%) | 51755 (23.8%) |
|  | 3-4 times per week | 38895 (22.1%) | 8857 (21.6%) | 47752 (22.0%) |
|  | Daily or almost daily | 41205 (23.4%) | 9360 (22.8%) | 50565 (23.3%) |
|  | Prefer not to answer | 168 (0.1%) | 44 (0.1%) | 212 (0.1%) |
|  | Missing | 147 (0.1%) | 203 (0.5%) | 350 (0.2%) |
| Physical activity level | Low (MET minutes ≤ 1200) | 47865 (27.2%) | 10673 (26.0%) | 58538 (26.9%) |
|  | High (MET minutes > 1200) | 91059 (51.7%) | 20333 (49.5%) | 111392 (51.3%) |
|  | Missing | 37325 (21.2%) | 10042 (24.5%) | 47367 (21.8%) |
| *APOE*-ε4 carrier status | Non-carrier | 125734 (73.7%) | 25899 (73.6%) | 151633 (73.7%) |
|  | Carrier | 44905 (26.3%) | 9310 (26.4%) | 54215 (26.3%) |
|  | Missing | 5610 (3.2%) | 5839 (14.2%) | 11449 (5.3%) |
| Waist circumference | Missing | 0 (0%) | 860 (2.1%) | 860 (0.4%) |
| Triglycerides | Missing | 0 (0%) | 14205 (34.6%) | 14205 (6.5%) |
| Blood pressure | Missing | 0 (0%) | 542 (1.3%) | 542 (0.2%) |
| HbA1c | Missing | 0 (0%) | 15121 (36.8%) | 15121 (7.0%) |
| HDL-cholesterol | Missing | 0 (0%) | 30850 (75.2%) | 30850 (14.2%) |
| Lipid-modifying medication | Yes | 48062 (27.3%) | 11076 (27.0%) | 59138 (27.2%) |
| Blood pressure medication | Yes | 60432 (34.3%) | 14048 (34.2%) | 74480 (34.3%) |
| Blood glucose medication | Yes | 8786 (5.0%) | 2217 (5.4%) | 11003 (5.1%) |
| *Abbreviations: PRS = Polygenic risk score, APOE = Apolipoprotein, SD = standard deviation, MET = metabolic equivalent of task, GBP = British pound sterling, HbA1c = hemoglobin A1c,* *HDL = high density lipoprotein.*  *Percentages do not add up to 100 due to rounding.* | | | | |

| Characteristic | | No Dementia  (N=170,994 ) | Dementia (N=5,255) | Overall (N=176,249) |
| --- | --- | --- | --- | --- |
| Age (years) | Mean (SD) | 64.1 (2.85) | 65.7 (2.71) | 64.1 (2.85) |
| Sex | Female | 89570 (52.4%) | 2482 (47.2%) | 92052 (52.2%) |
|  | Male | 81424 (47.6%) | 2773 (52.8%) | 84197 (47.8%) |
| Ethnicity | White | 165627 (96.9%) | 5047 (96.0%) | 170674 (96.8%) |
|  | Non-White | 4590 (2.7%) | 174 (3.3%) | 4764 (2.7%) |
|  | Missing | 777 (0.5%) | 34 (0.6%) | 811 (0.5%) |
| Education level | Primary | 45328 (26.5%) | 1897 (36.1%) | 47225 (26.8%) |
|  | Secondary | 75752 (44.3%) | 1933 (36.8%) | 77685 (44.1%) |
|  | Post-secondary non-tertiary | 18405 (10.8%) | 517 (9.8%) | 18922 (10.7%) |
|  | Tertiary | 29224 (17.1%) | 797 (15.2%) | 30021 (17.0%) |
|  | Missing | 2285 (1.3%) | 111 (2.1%) | 2396 (1.4%) |
| Townsend deprivation index, quintiles | 1 (least deprived) | 34294 (20.1%) | 927 (17.6%) | 35221 (20.0%) |
|  | 2 | 34284 (20.0%) | 936 (17.8%) | 35220 (20.0%) |
|  | 3 | 34258 (20.0%) | 962 (18.3%) | 35220 (20.0%) |
|  | 4 | 34171 (20.0%) | 1049 (20.0%) | 35220 (20.0%) |
|  | 5 (most deprived) | 33846 (19.8%) | 1374 (26.1%) | 35220 (20.0%) |
|  | Missing | 141 (0.1%) | 7 (0.1%) | 148 (0.1%) |
| Household income (in GBP) | Less than 18,000 | 46821 (27.4%) | 1864 (35.5%) | 48685 (27.6%) |
|  | 18,000 to 30,999 | 45362 (26.5%) | 1268 (24.1%) | 46630 (26.5%) |
|  | 31,000 to 51,999 | 29395 (17.2%) | 541 (10.3%) | 29936 (17.0%) |
|  | 52,000 to 100,000 | 14226 (8.3%) | 211 (4.0%) | 14437 (8.2%) |
|  | Greater than 100,000 | 3354 (2.0%) | 55 (1.0%) | 3409 (1.9%) |
|  | Missing | 31836 (18.6%) | 1316 (25.0%) | 33152 (18.8%) |
| Smoking status | Never | 84921 (49.7%) | 2364 (45.0%) | 87285 (49.5%) |
|  | Previous | 71056 (41.6%) | 2336 (44.5%) | 73392 (41.6%) |
|  | Current | 14011 (8.2%) | 497 (9.5%) | 14508 (8.2%) |
|  | Missing | 1006 (0.6%) | 58 (1.1%) | 1064 (0.6%) |
| Alcohol intake | Never | 8044 (4.7%) | 386 (7.3%) | 8430 (4.8%) |
|  | Former drinker | 6291 (3.7%) | 351 (6.7%) | 6642 (3.8%) |
|  | Special occasions only | 20538 (12.0%) | 745 (14.2%) | 21283 (12.1%) |
|  | 1-3 times per month | 16863 (9.9%) | 538 (10.2%) | 17401 (9.9%) |
|  | 1-2 times per week | 40932 (23.9%) | 1146 (21.8%) | 42078 (23.9%) |
|  | 3-4 times per week | 37913 (22.2%) | 982 (18.7%) | 38895 (22.1%) |
|  | Daily or almost daily | 40118 (23.5%) | 1087 (20.7%) | 41205 (23.4%) |
|  | Prefer not to answer | 159 (0.1%) | 9 (0.2%) | 168 (0.1%) |
|  | Missing | 136 (0.1%) | 11 (0.2%) | 147 (0.1%) |
| Physical activity level | Low (MET minutes ≤ 1200) | 46436 (27.2%) | 1429 (27.2%) | 47865 (27.2%) |
|  | High (MET minutes > 1200) | 88584 (51.8%) | 2475 (47.1%) | 91059 (51.7%) |
|  | Missing | 35974 (21.0%) | 1351 (25.7%) | 37325 (21.2%) |
| *APOE*-ε4 carrier status | Non-carrier | 123352 (72.1%) | 2382 (45.3%) | 125734 (71.3%) |
|  | Carrier | 42220 (24.7%) | 2685 (51.1%) | 44905 (25.5%) |
|  | Missing | 5422 (3.2%) | 188 (3.6%) | 5610 (3.2%) |
| Elevated waist circumference | Present | 63888 (37.3%) | 2118 (40.3%) | 66066 (37.5%) |
| Elevated triglycerides | Present | 74819 (43.8%) | 2277 (43.3%) | 77096 (43.7%) |
| Elevated blood pressure* | Present | 143196 (83.7%) | 4623 (88.0%) | 147819 (83.9%) |
| Elevated HbA1c* | Present | 42774 (25.0%) | 1757 (33.4%) | 44531 (25.3%) |
| Reduced HDL-cholesterol* | Present | 66106 (38.7%) | 2573 (49.0%) | 68679 (39.0%) |
| *Abbreviations: PRS = Polygenic risk score, APOE = Apolipoprotein, SD = standard deviation, MET = metabolic equivalent of task, GBP = British pound sterling, HbA1c = hemoglobin A1c,* *HDL = high density lipoprotein.*  *Percentages do not add up to 100 due to rounding.*  **Includes medication use* | | | | |

**Supplemental File 7: Baseline characteristics according to incident dementia status at the end of follow-up**

**Supplemental File 8: Effect of individual and sequential adjustment of covariates among Cox proportional hazards model investigating the association between MetS and incident dementia**

*A) Individual adjustment*

*B) Sequential adjustment*

**Supplemental File 9: Sensitivity analyses**

| Sensitivity Analysis Performed | MetS | Cases/Population | HR (95%CI) |
| --- | --- | --- | --- |
| Age as a time scale | No | 2676 / 102,739 | Ref. |
|  | Yes | 2579 / 73,510 | 1.12 (1.07, 1.18) |
|  | | | |
| Excluding Participants in Wales | No | 2644 / 98,806 | Ref. |
|  | Yes | 2556 / 70,229 | 1.13 (1.07, 1.19) |
|  | | | |
| Additional adjustment for CVD | No | 2676 / 102,739 | Ref. |
|  | Yes | 2579 / 73,510 | 1.05 (1.01, 1.13) |
|  | | | |
| Treating death as a competing risk | No | 2676 / 102,739 | Ref. |
|  | Yes | 2579 / 73,510 | 1.09 (1.04, 1.16) |
|  | | | |
| Using NCEP-ATP III criteria to define MetS | No | 3012 / 109,739 | Ref. |
|  | Yes | 2243 / 66,510 | 1.10 (1.03, 1.16) |
|  | | | |
| Multiple Imputation | No | 3348 / 126,471 | Ref. |
|  | Yes | 3240 / 90,826 | 1.11 (1.06, 1.17) |
| *CVD = Cardiovascular disease; NCEP-ATP III = National Cholesterol Education Program – Adult Treatment Panel III; MetS = Metabolic syndrome*  *Models adjusted for age, sex, ethnicity, Townsend deprivation index, education, household income, smoking status, alcohol intake, physical activity, APOE-ε4 carrier status.* | | | |

**Supplemental File 10: Cox proportional hazards models investigating the association between MetS and incident dementia according to sex**

| Sex | Cases/Population | HR (95%CI) |
| --- | --- | --- |
| Female | | |
| No MetS | 1337 / 56,323 | Ref. |
| MetS | 1145 / 35,729 | 1.08 (1.01, 1.17) |
| Male | | |
| No MetS | 1339 / 46, 416 | Ref. |
| MetS | 1434 / 37,781 | 1.16 (1.07, 1.25) |
| *Abbreviations: HR = Hazard Ratio, CI = Confidence Interval, , Ref. = Reference group, APOE = Apolipoprotein E, MetS = Metabolic syndrome*  *Models adjusted for age, ethnicity, Townsend deprivation index, education, household income, smoking status, alcohol intake, physical activity, APOE-ε4 carrier status. P-value for overall interaction between MetS and sex: 0.33.* | | |

**Supplemental File 11: Cox proportional hazards models investigating the association between MetS and incident dementia among different age groups**

| Age Grouping | Cases/Population | HR (95%CI) |
| --- | --- | --- |
| Inclusion of All Participants Aged ≥50 Years | | |
| No MetS | 3073 / 196,298 | Ref. |
| MetS | 2860 / 114,719 | 1.13 (1.07, 1.19) |
|  |  |  |
| Age-Stratification | | |
| <60 Years |  |  |
| No MetS | 397 / 93,559 | Ref. |
| MetS | 281 / 41,209 | 1.22 (1.04, 1.43) |
| 60 to <65 Years |  |  |
| No MetS | 801 / 38,277 | Ref. |
| MetS | 1781 / 42,512 | 1.19 (1.08, 1.32) |
| ≥65 Years |  |  |
| No MetS | 1781 / 42,512 | Ref. |
| MetS | 1778 / 35,233 | 1.08 (1.01, 1.16) |
| *Abbreviations: HR = Hazard Ratio, CI = Confidence Interval, , Ref. = Reference group, PRS = Polygenic risk score, APOE = Apolipoprotein E, MetS = Metabolic syndrome*  *Models adjusted for age, sex, ethnicity, Townsend deprivation index, education, household income, smoking status, alcohol intake, physical activity, APOE-ε4 carrier status.* | | |

**Supplemental File 12: 12-year risk of incident dementia by MetS status and genetic predisposition for dementia**

**Supplemental File 13: Joint associations of A) MetS and *APOE* ε4 carrier status and B) MetS and non-*APOE* Dementia PRS for risk of incident dementia**

| Genetic Factor | Cases/Population | HR (95%CI) | 12-Year Cumulative Incidence (95%CI) |
| --- | --- | --- | --- |
| *APOE* ε4 | | | |
| *APOE* ε4 - / MetS - | 1152 / 73,825 | 1 (Ref.) | 1.40 (1.30, 1.50) |
| *APOE* ε4 - / MetS + | 1230 / 51,909 | 1.28 (1.18, 1.39) | 2.30 (2.20, 2.40) |
| *APOE* ε4 + / MetS - | 1424 / 25,686 | 3.70 (3.42, 4.02) | 5.10 (4.80, 5.40) |
| *APOE* ε4 + / MetS + | 1261 / 19,219 | 3.71 (3.43, 4.01) | 6.50 (6.20, 6.90) |
|  |  |  |  |
| Non-*APOE* Dementia PRS |  |  |  |
| Low PRS / MetS - | 297 / 16,759 | 1 (Ref.) | 1.70 (1.50, 1.90) |
| Low PRS / MetS + | 337 / 12,255 | 1.29 (1.10, 1.51) | 2.80 (2.50, 3.10) |
| Intermediate PRS / MetS - | 1272 / 50,852 | 1.42 (1.25, 1.61) | 2.30 (2.20, 2.50) |
| Intermediate PRS / MetS + | 1225 / 36,188 | 1.60 (1.41, 1.82) | 3.30 (3.20, 3.50) |
| High PRS / MetS - | 594 / 17,159 | 1.98 (1.72, 2.28) | 3.20 (2.90, 3.50) |
| High PRS / MetS + | 557 / 11,854 | 2.30 (2.00, 2.65) | 4.60 (4.20, 5.00) |
| *HR = Hazard Ratio, CI = Confidence Interval, , Ref. = Reference group, PRS = Polygenic risk score, APOE = Apolipoprotein, MetS = Metabolic syndrome*  *A) Model adjusted for age, sex, ethnicity, Townsend deprivation index, education, household income, smoking status, alcohol intake, physical activity. Excluded: 5,610 with missing information on APOE ε4 carrier status. P-value for overall interaction between MetS and APOE ε4: <0.001*  *B) Model adjusted for age, sex, ethnicity, Townsend deprivation index, education, household income, smoking status, alcohol intake, physical activity, APOE ε4 carrier status. Excluded: 31,182 with missing information on non-APOE dementia PRS. P-value for overall interaction between MetS and non-APOE dementia PRS: 0.42* | | | |

**Supplemental File 14: Cox proportional hazards models investigating the association between individual MetS components and incident dementia according to varying follow-up length**

*HR = Hazard Ratio, CI = Confidence Interval, WC: Elevated Waist Circumference, HDL: Reduced High-Density Lipoprotein, TG: Elevated Triglycerides, BP: Elevated Blood Pressure, HbA1c: Elevated HbA1c*

*Models adjusted for age, sex, ethnicity, Townsend deprivation index, education, household income, smoking status, alcohol intake, physical activity, APOE-ε4 carrier status.*

**Follow-up length: 10+ years**

**Follow-up length: 0-5 years**

**Follow-up length: >5 - 10 years**

**Supplemental File 15: Cox proportional hazards model investigating the association between all possible MetS combinations and incident dementia**

*HR = Hazard Ratio, CI = Confidence Interval, WC: Elevated Waist Circumference, HDL: Reduced High-Density Lipoprotein, TG: Elevated Triglycerides, BP: Elevated Blood Pressure, HbA1c: Elevated HbA1c*

*Model adjusted for age, sex, ethnicity, Townsend deprivation index, education, household income, smoking status, alcohol intake, physical activity, APOE-ε4 carrier status.*

**Supplemental File 16: Cox proportional hazards models investigating the association between the number of MetS components and incident dementia according to varying follow-up length**

**
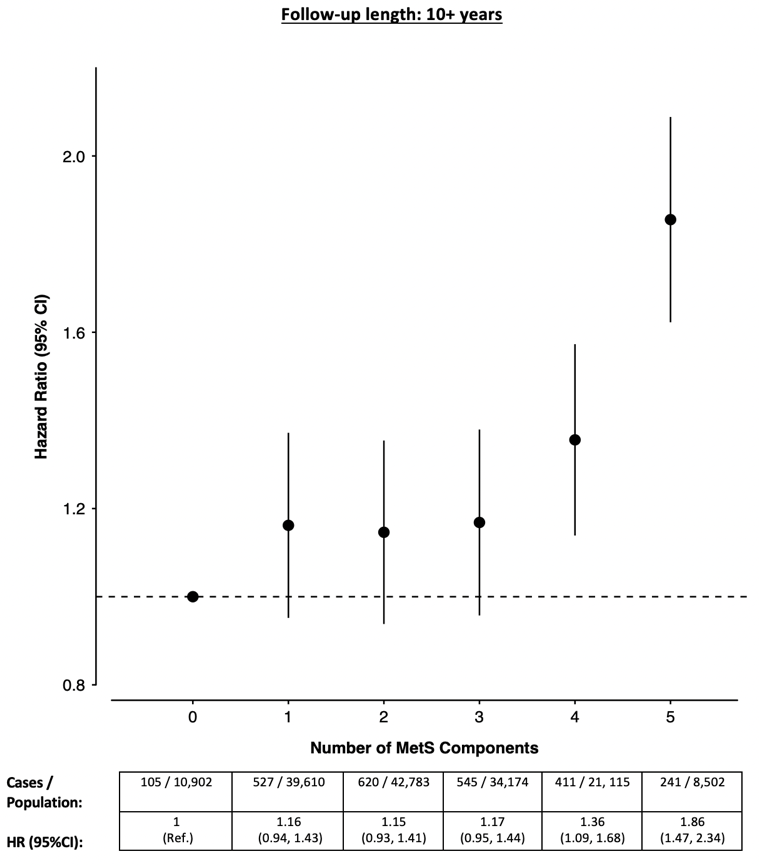

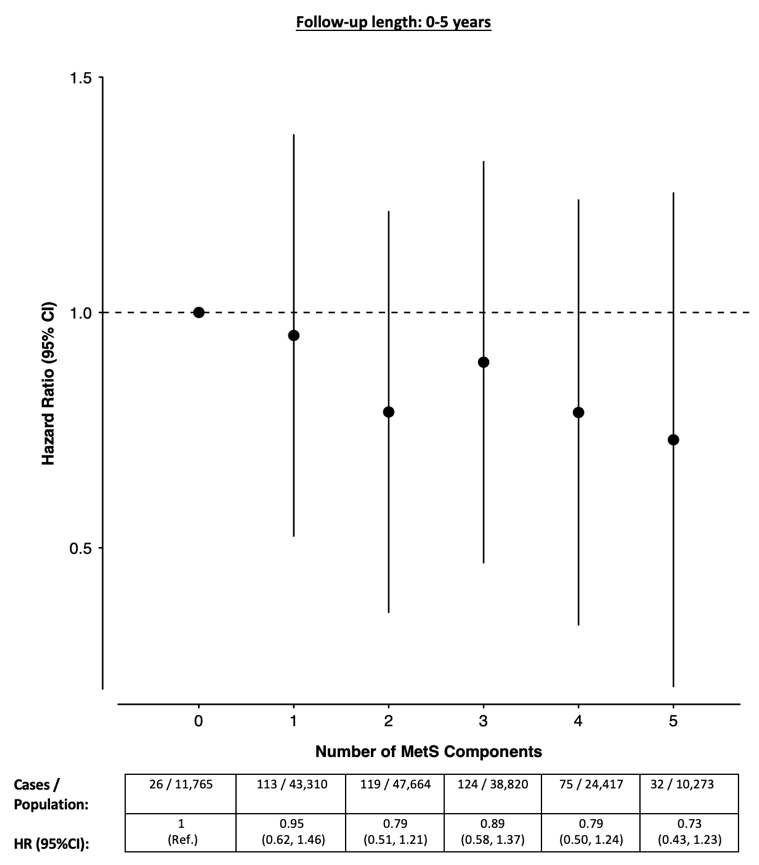

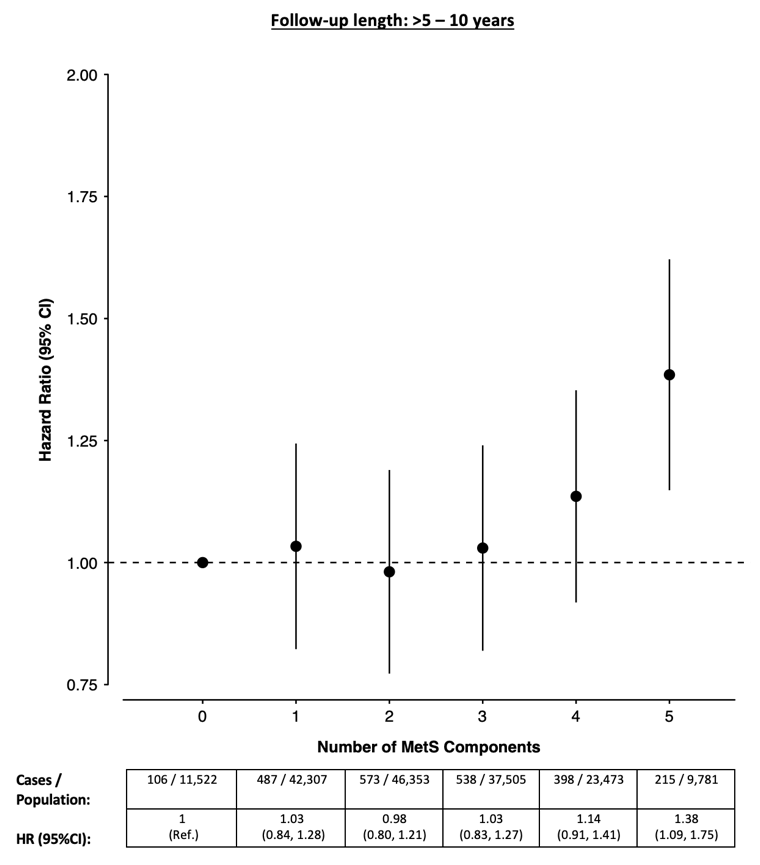
**

*HR = Hazard Ratio, CI = Confidence Interval*

*Models adjusted for age, sex, ethnicity, Townsend deprivation index, education, household income, smoking status, alcohol intake, physical activity, APOE-ε4 carrier status.*
